# Experimental study on crack characteristics and acoustic emission characteristics in rock-like material with pre-existing cracks

**DOI:** 10.1038/s41598-021-03162-6

**Published:** 2021-12-10

**Authors:** Ji-Shun Pan, Shuang-Xi Yuan, Tong Jiang, Cheng-Hao Cui

**Affiliations:** College of Geosciences and Engineering, North China University of Water Resources and Electric Power, Zhengzhou, 450046 China

**Keywords:** Geology, Civil engineering

## Abstract

Grain size composition, crack pattern, and crack length have a significant influence on the crack characteristics, mechanical characteristics, and acoustic emission characteristics of rock masses. In this paper, the crack characteristics, mechanical characteristics, and acoustic emission characteristics of rock masses with different grain size compositions, different crack patterns, and different crack lengths were investigated under uniaxial compression. The rock masses were made of rock-like materials. The crack initiation locations and crack propagation directions were different for a specimen comprised of one grain size range compared with specimens comprised of two or three grain size ranges. The specimens comprised of one and three grain size ranges crack progressively. The specimen comprised of two-grain size ranges brittle fracture. The highest peak axial load was found in the specimens comprised of one grain size range. The results showed that tensile wing crack, anti-tensile wing crack, transverse shear crack, compression induced tensile crack, and surface spalling were produced in specimens with different crack orientations. The rock mass with 2 cm long crack started to produce cracks from the tip of the crack extending to the top and bottom surface, soon forming through cracks. The rock was brittle fracture. The axial load reached the maximum and then fell rapidly. The acoustic emission energy reached a rapid maximum and then decreased rapidly. The rock mass with 3 cm long fissures started to produce cracks that only extended from the tip of the fissures to the top surface but not to the bottom surface. The rock mass was progressively fractured. The axial load was progressively decreasing. The acoustic emission energy also rose and fell rapidly several times as the rock mass was progressively fractured. Different rock crack lengths led to different crack processes and crack patterns, resulting in very different acoustic emission characteristics.

## Introduction

Joints, fissures and cracks of varying size and orientation are widely distributed throughout the rock mass. These have a tremendous influence on the mechanical properties and damage characteristics of the rock mass. A great deal of research has been done by previous authors to reveal the influence of joints and fissures on the mechanical characteristics and fracture characteristics of rock masses, and fruitful results have been achieved. They adopted the research methods of physical model tests, numerical simulations and theoretical analysis. For the model test studies they have used uniaxial compression, direct shear and triaxial compression.

Using model experiments can get data close to the prototype. Over the years, researchers have published relevant research on the properties of jointed rock. The mechanical behaviour of sandstones with closed fissures was investigated by conventional triaxial compression tests and numerical simulations by Huang et al.^1^.

The results showed that the stress–strain curves of the rock exhibited a Z-shaped characteristic and a bimodal stress trend. Gehle and Kutter investigated the damage characteristics of this fractured rock mass through shear tests on multi-fractured specimens made of gypsum^2^. The results of the study showed that the specimens fractured in three stages: (1) Tensile fracture. (2) Rolling and sliding friction in the swollen joint zone. (3) Sliding within a joint fill consisting of fractured material. Using uniaxial compression and a particle flow code, Cao et al. investigated the effect of openings and joints on the crack growth behaviour around openings^3^. By means of a prefabricated physical model, Fan et al. analysed the damage pattern and fracture evolution of rock specimens with joints and openings under uniaxial compression^4,5^. They further investigated how the inclination angle of the joint affected the distribution of principal stresses around the opening. By performing triaxial compression tests on the jointed rock masses and analysing the stress–strain curves of the rock masses, Wang obtained the cohesion and internal friction angle of the joint surface at M-C strength^6^. Xiao et al. carried out uniaxial compression tests on model gypsum with different inclination angles β^7^. They obtained many parameters of the rock mass, such as joint strength. By performing several uniaxial compression tests on the jointed rock mass and gypsum, Kulatilake et al. understood the mechanical properties of the rock mass and understood the effect of the relationship between joints on the strength of the rock mass^8^. Carpinteri et al. carried out three-point bending tests and analysed acoustic emission activity and energy fields^9^. The classification of cracking modes could be carried out. It was found that the fracture energy and acoustic emission energy varied inversely with the size of the specimen.

Numerical simulation can be used to study the properties of fractured rock masses from multiple angles, at low cost, and throughout the process. Several numerical simulations were carried out by UDEC. Vergara et al. explored how parallel non-persistent joints affected the mechanical behaviour of the rock mass^10^. The results showed that a change in joint orientation will lead to a large anisotropy in the strength of the rock mass. Through uniaxial compression of blocks with multiple non-persistent joints, their mechanical behaviour was investigated using PFC3D by Fan et al.^11^. They also investigated how particle size, stiffness and joint strength parameters affected the deformability and damaged patterns of the specimens. By developing a smooth nodular model, Potyondy and Cundall had this new approach to study nodular rocks^12^. The model reproduced many features of rock behavior, including elasticity, fracturing, acoustic emission, damage accumulation producing material anisotropy, hysteresis, dilation and post-peak softening. By extending the smooth joint model, Ivars et al. studied the strength of jointed rock masses^13^. Carpinteri et al. proposed an analytical/numerical model which was called overlapping crack model for the analysis of the mechanical behaviour of concrete in compression^14^. The overlapping crack model was introduced into the finite element method. Numerical simulations of eccentric compression tests were carried out and compared with the test results. The influence of the size-scale, the specimen slenderness and the degree of load eccentricity were investigated. The influence of each parameter on the ductility characteristics of the rock mass was quantified.

The mechanical characteristics, fracture characteristics, fracture processes, crack extension, fracture modes, stress distribution, acoustic emission characteristics, mechanical behaviour and strength characteristics of fractured rock masses have been studied by previous authors through model tests and numerical simulations. Fruitful results were achieved. These studies reveal the effects of fracturing on the mechanical characteristics, fracture characteristics, acoustic emission characteristics and strength characteristics of rock masses. They contribute to our understanding of the role and hazards of fractures.

The effects of fractures on the strength characteristics, fracture characteristics, fracture modes, deformation characteristics and stress variation characteristics of rock masses under different forms of forces were understood from the point of view of model tests. From the point of view of numerical simulation, new and old numerical models were applied to gain an understanding of the effects of fractures on the strength characteristics, damage characteristics, deformation characteristics, mechanical behaviour, damage modes and ductility characteristics of the rock mass.

The above studies have provided quite useful information. However, there are fewer studies on the mechanical characteristics, damage characteristics, acoustic emission characteristics and crack extension processes of square rock masses with different grain size compositions and different modes of edge fracture rock masses. It would also be interesting to study the crack extension sequence in conjunction with changes in acoustic emission characteristics and stress changes.

Uniaxial compression was used to study the fracturing, acoustic emission and mechanical properties of fractured material with different crack modes, different grain sizes and different crack lengths in this paper.

## Test procedures

### Sample preparation

**Figure 1:**
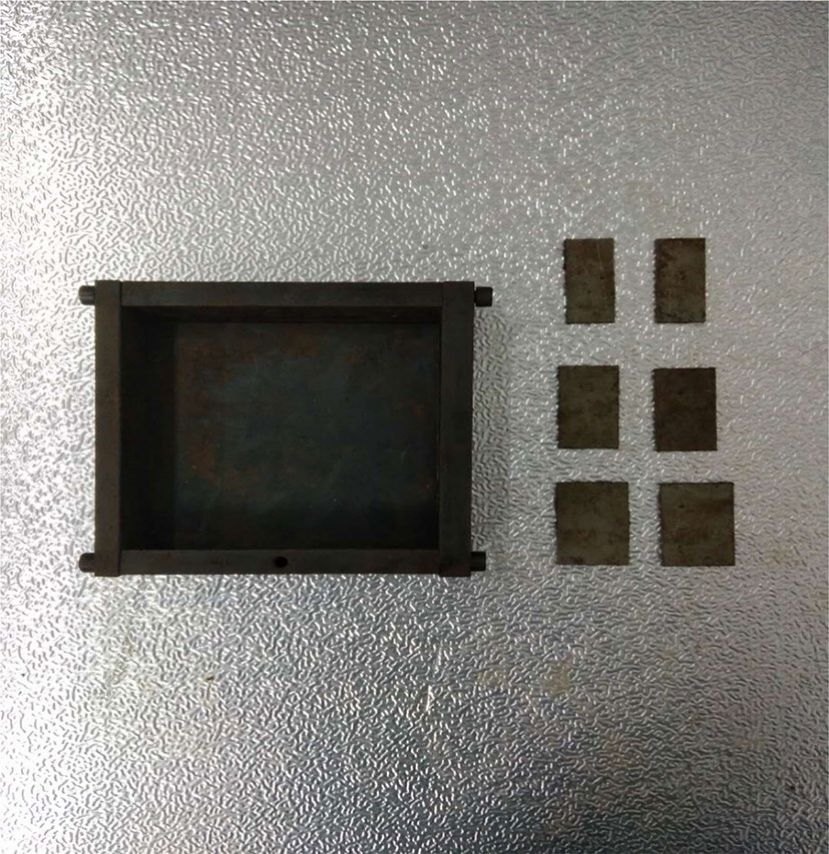
Mould for casting samples and six steel sheets used to form cracks in the samples.

**Figure 2:**
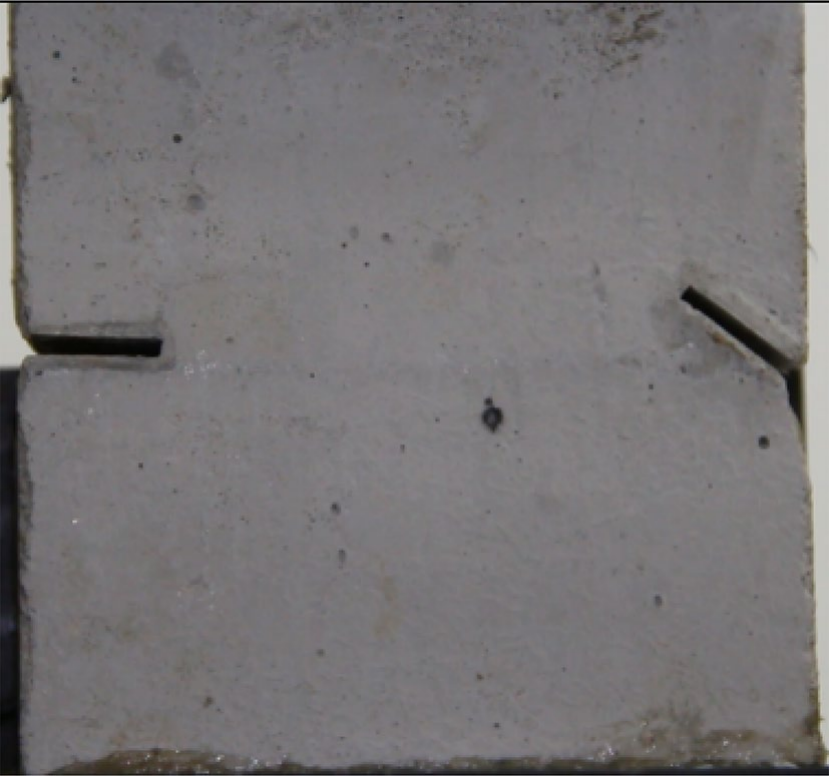
Finished sample before testing.

The tested specimens were 120 × 120 × 30 mm in size. The tested specimens were comprised of C425 cement, sand, and water in a weight ratio of 1:1:0.7. The specimens also contained a rapid setting agent. The sand was sieved to three particle sizes, 0–0.6 mm, 0.6–1 mm and 1–2.56 mm. Crack were cast into each specimen and the crack were formed by inserting a steel sheet into the cement–sand–water mix before the specimen solidified in the mould. The steel sheets were 45 mm long; 20, 25 or 30 mm wide; and 2.5 mm thick. The specimen preparation procedure was as follows. First, equal weights of cement and sand were mixed and stirred evenly, then the appropriate amount of water was added. After the components were mixed again, the steel sheets were placed at the proper positions and angles in the mould. After that, the mould was filled with the cement mortar mix layer by layer. Each specimen remained in its mould for 24 h to harden before it was taken out of the mould. The specimens were then cured for 15 days (watered frequently) before testing. The moulds for making the samples were shown in Fig. 1. The test specimen was shown in Fig. 2.

To study how different crack orientations affected the specimens’ fracturing, specimens with different crack orientations were prepared. To study the effects of crack length on fracturing and acoustic emission characteristics of specimens, specimens with different crack lengths, 20 mm and 30 mm, were prepared. To study the effect of different particle sizes on fracturing, and acoustic emission characteristics of specimens, specimens were prepared with three different sand particle sizes. The first tested specimens were prepared with 1–2.56 mm sand, bimodal specimens prepared with 0–0.6 mm and 1–2.56 mm sand, and a third specimen prepared with 0–0.6 mm, 0.6–1.0 mm, and 1–2.56 mm sand. This suite of specimens represented fractured rocks with different degrees of non-homogeneity.

### Test methods

**Figure 3:**
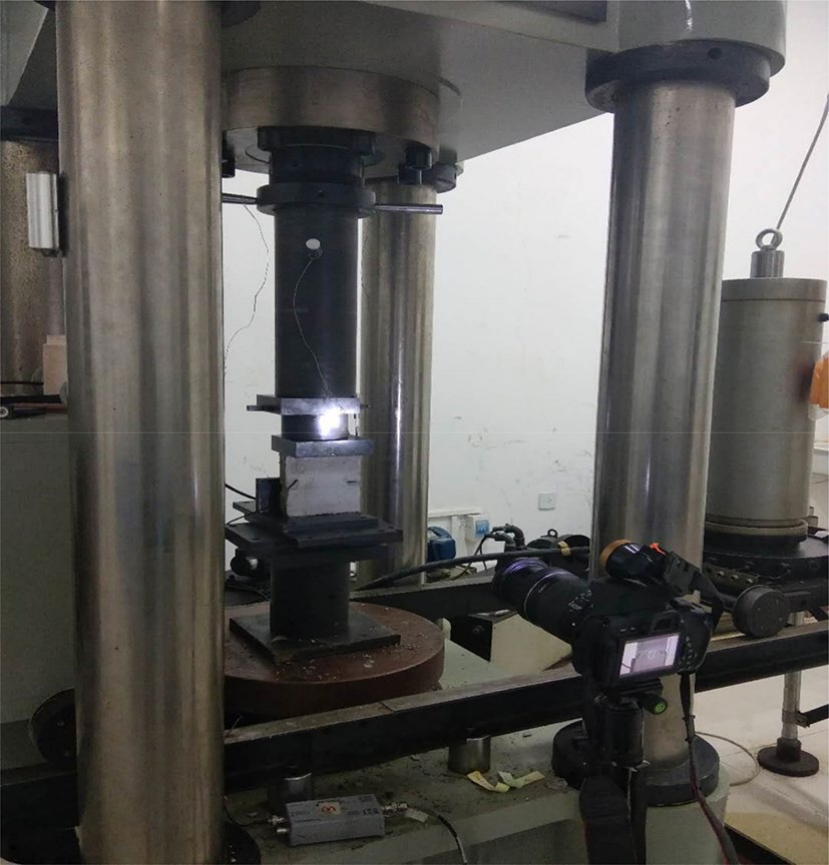
Photograph of the uniaxial press showing a specimen ready to be tested.

The top and bottom surfaces of each specimen were coated with petroleum jelly to reduce end effects. The same axial displacement rate, 0.9 mm/min, was used for each uniaxial compression test. An acoustic emissions monitor was used to record the acoustic emissions during rock fracturing. A high-definition camera was used to film crack propagation. Both the displacement and the axial load during the tests were recorded. The servo press, camera, and acoustic emission monitor were turned on to record data at the same time. The test method and equipment are shown in Fig. 3.

## Experimental results and analysis

### Crack orientations and their influence on fracturing

**Figure 4:**
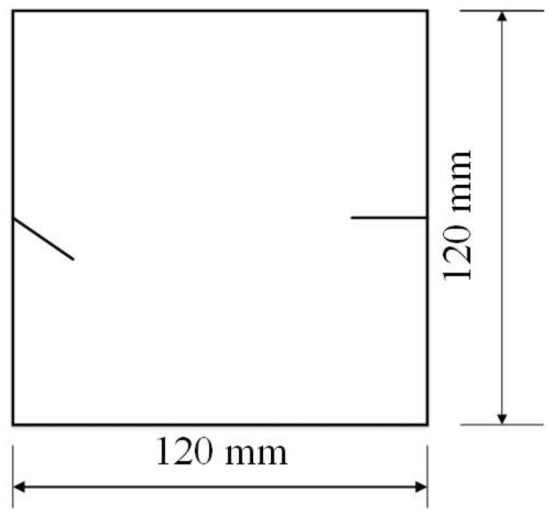
Crack orientation 1.

**Figure 5:**
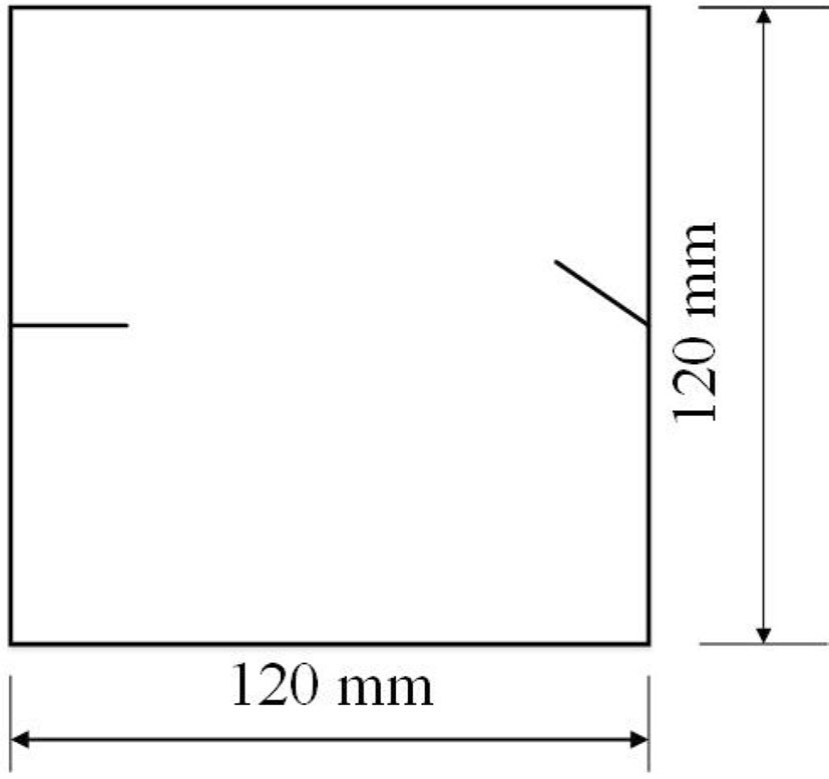
Crack orientation 2.

**Figure 6:**
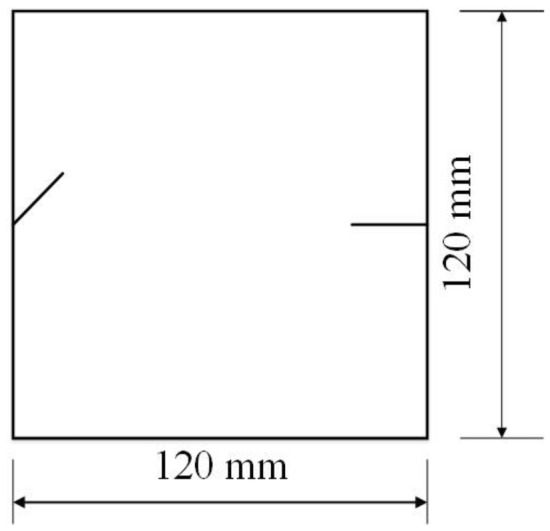
Crack orientation 3.

**Figure 7:**
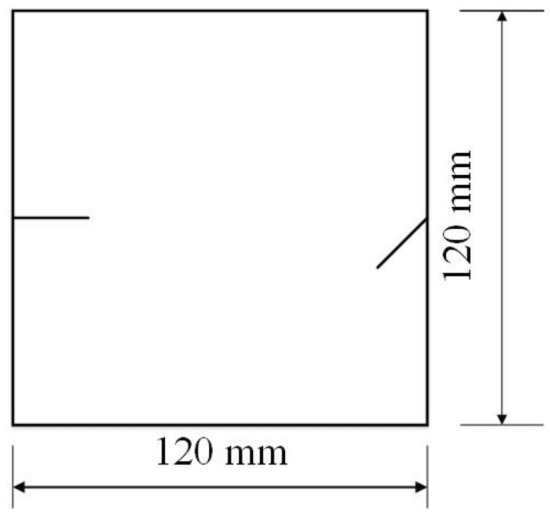
Crack orientation 4.

To study how crack orientations affected rocks under uniaxial compression, four different crack orientations were tested. These orientations are shown in Figs. 4, 5, 6 and 7.

**Figure 8:**
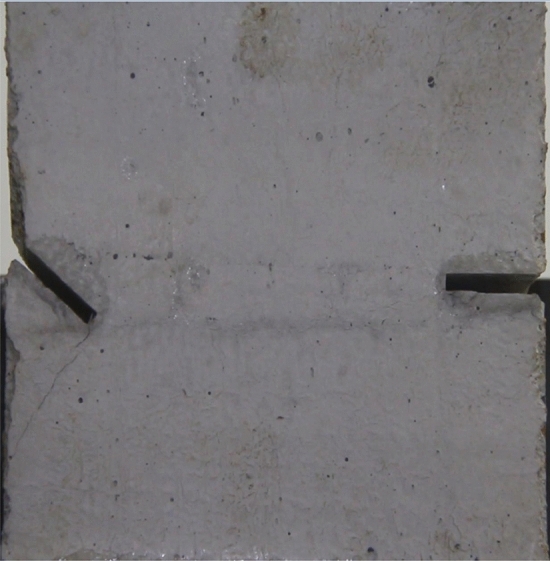
Photograph of a specimen showing of tensile wing crack appearing.

**Figure 9:**
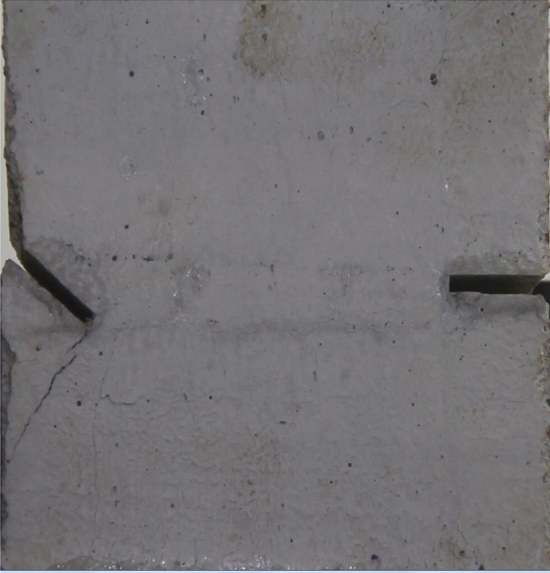
Photograph of a specimen showing tensile wing crack propagation.

**Figure 10:**
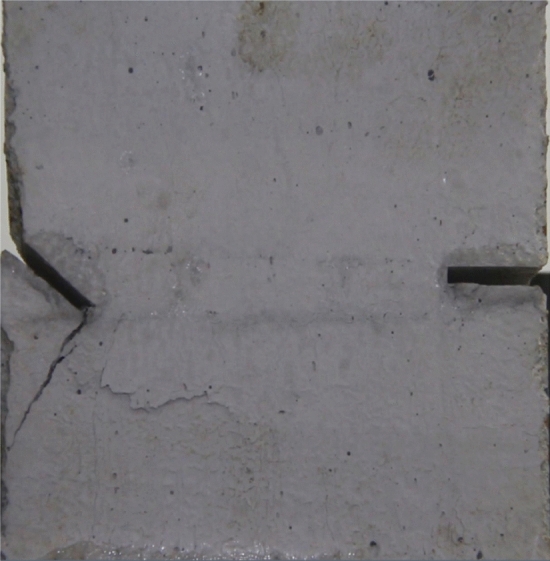
Photograph of a specimen showing transverse shear crack appearing.

**Figure 11:**
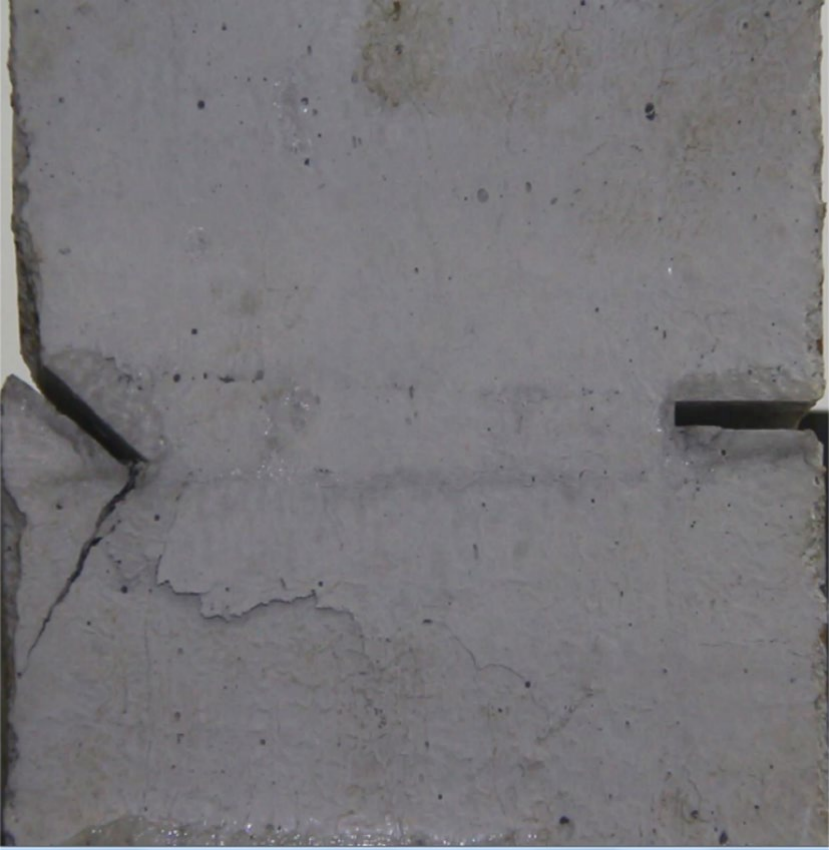
Photograph of a specimen showing transverse shear crack propagation.

**Figure 12:**
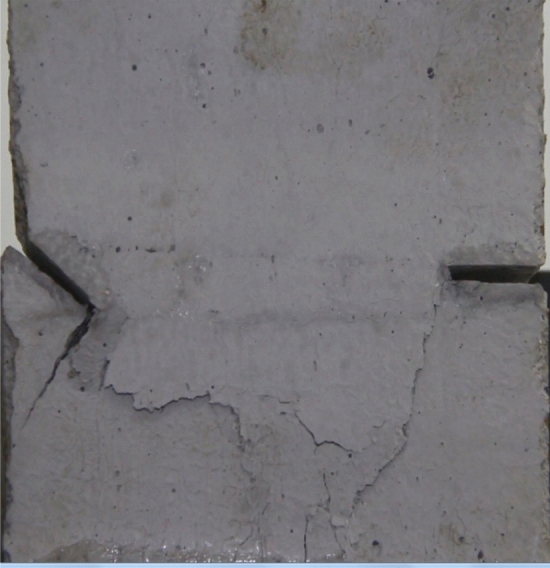
Photograph of a specimen showing propagated tensile wing crack.

**Figure 13:**
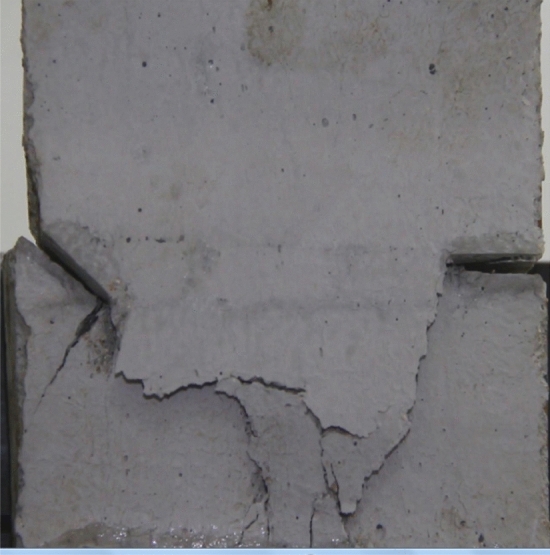
Photograph of a specimen showing crack penetration.

Figures 8, 9, 10, 11, 12 and 13 show photographs of a specimen with orientation 1 cracks rupture process under uniaxial compression.

As can be seen from Figs. 8, 9, 10, 11, 12 and 13. The first was a tensile wing crack extending from the tip of the left-hand fissure to the left boundary. As the axial load increased, a transverse shear crack was produced in the middle of the rock mass crack, then a downward extending tensile wing crack from the tip of the fissure on the right side of the rock mass was produced.

**Figure 14:**
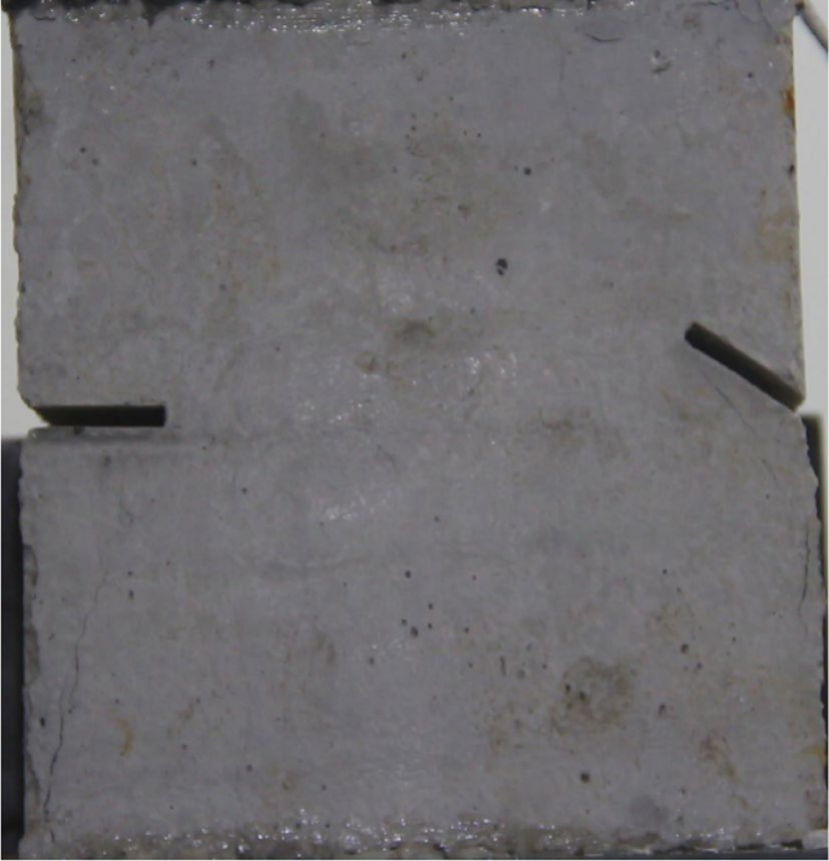
Photograph of a fractured specimen showing tensile crack from the bottom surface caused by compression and anti-tensile wing crack appearing.

**Figure 15:**
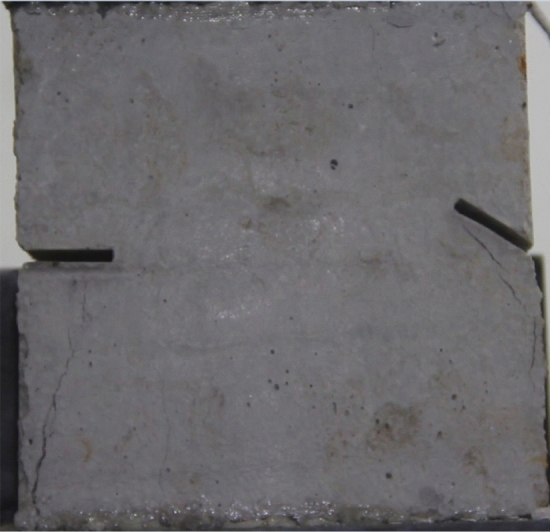
Photograph of a specimen showing tensile crack from the bottom surface caused by compression and anti-tensile wing crack propagation.

**Figure 16:**
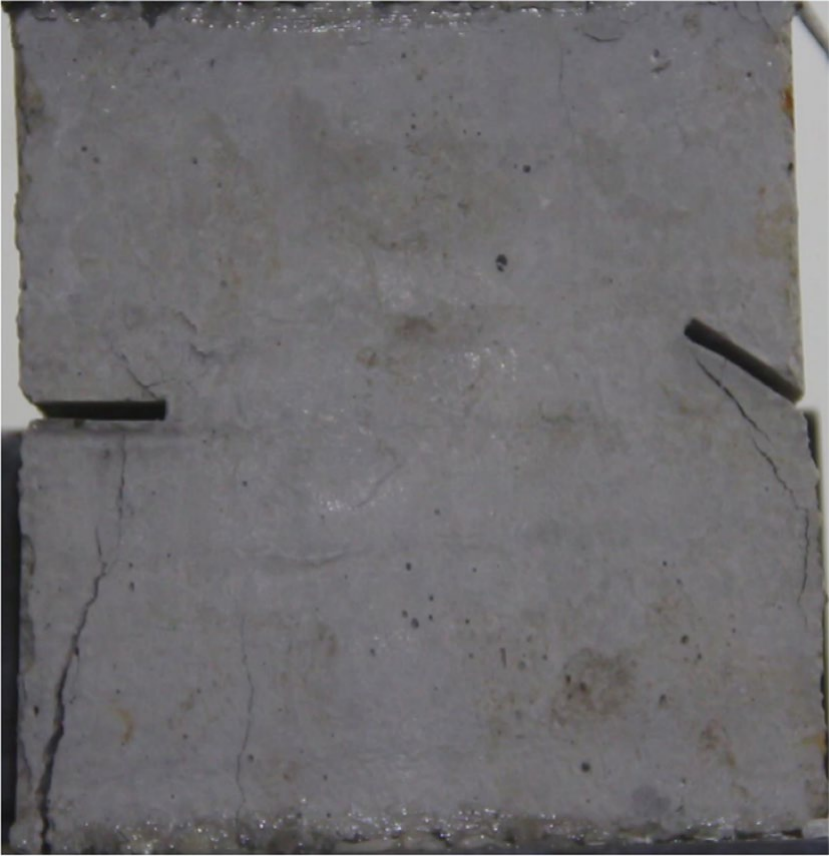
Photograph of a specimen showing another set of anti-tensile wing crack appearing.

**Figure 17:**
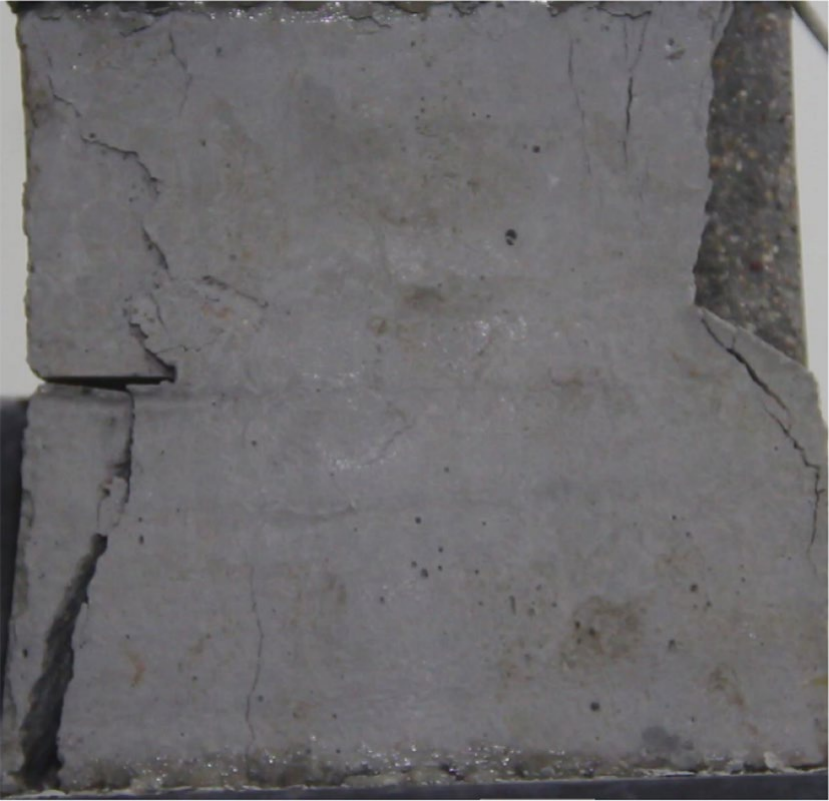
Photograph of a specimen showing tensile crack from the top surface caused by compression propagating.

Figures 14, 15, 16 and 17 show photographs of a specimen with orientation 2 cracks rupture process under uniaxial compression.

As can be seen from Figs. 14, 15, 16 and 17. The first was a tensile crack extending from the bottom of the left side of the rock mass toward the fissure. As the axial load increased, a anti-tensile wing crack from the tip of the right fissure towards the right boundary was produced, then a tensile crack from the tip of the left-hand fissure towards the top surface of the rock mass was produced. After that the tensile crack from the tip of the right-hand fissure towards the top surface was produced.

**Figure 18:**
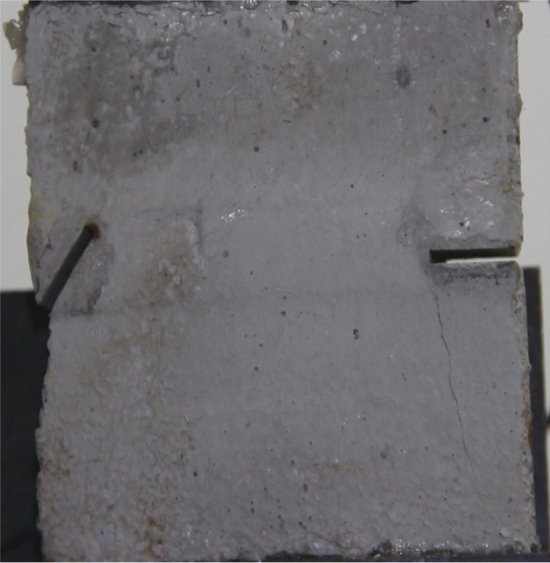
Photograph of a specimen showing tensile wing crack appearing.

**Figure 19:**
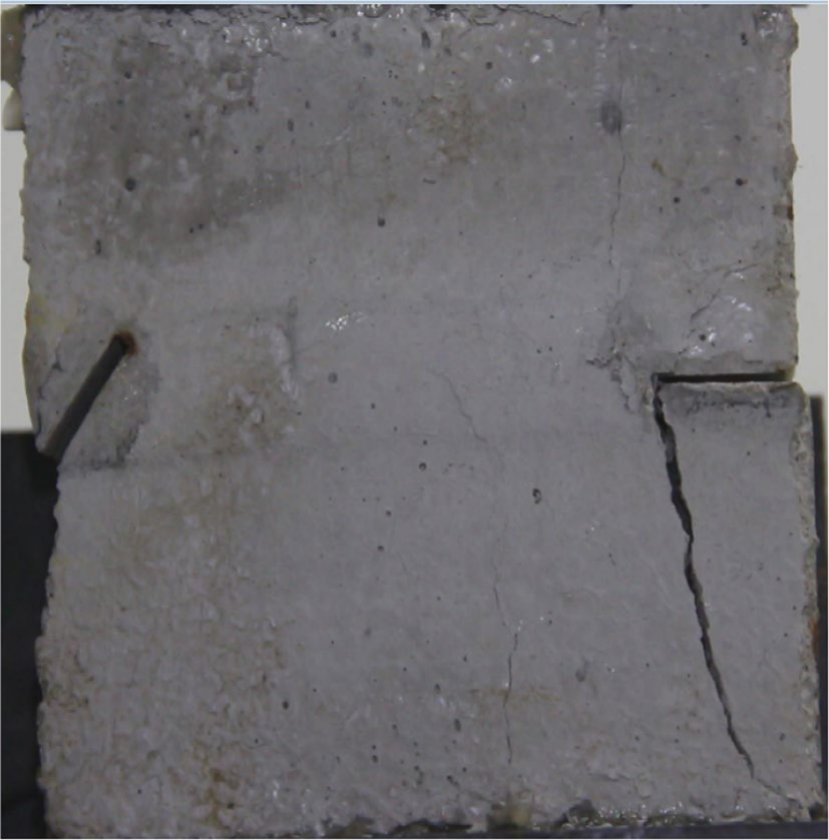
Photograph of a specimen showing tensile crack from the top surface caused by compression appearing and tensile wing crack propagation.

**Figure 20:**
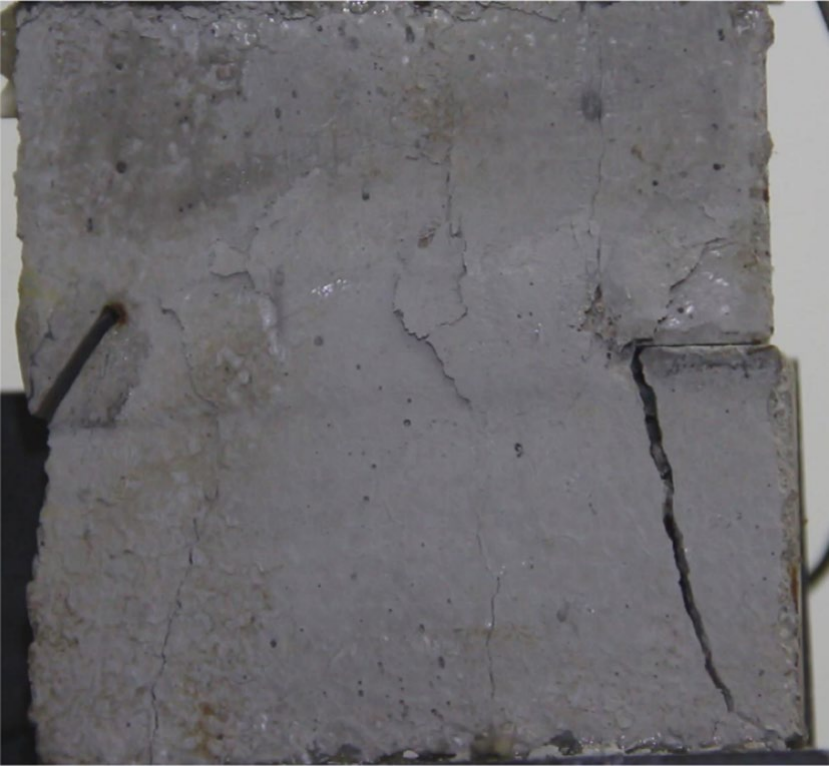
Photograph of a specimen showing another tensile crack from the top surface caused by compression appearing.

**Figure 21:**
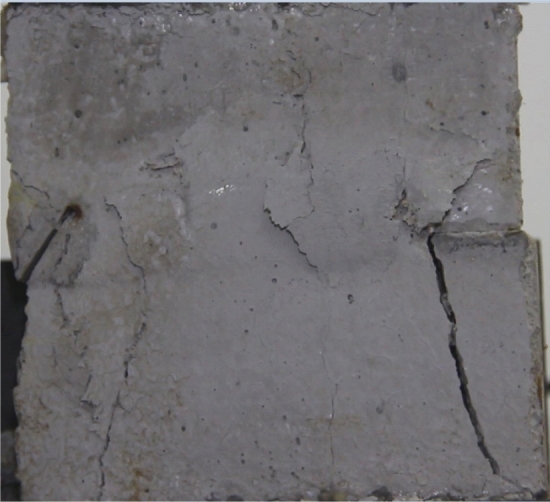
Photograph of a specimen showing an anti-tensile wing crack appearing.

**Figure 22:**
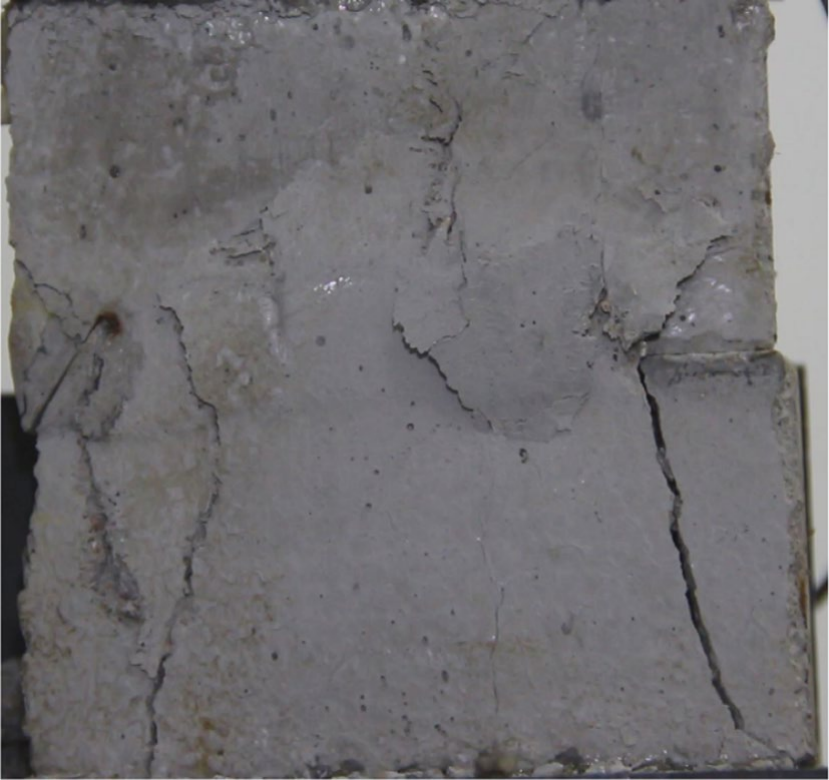
Photograph of a specimen showing tensile crack from the top surface caused by compression propagating.

**Figure 23:**
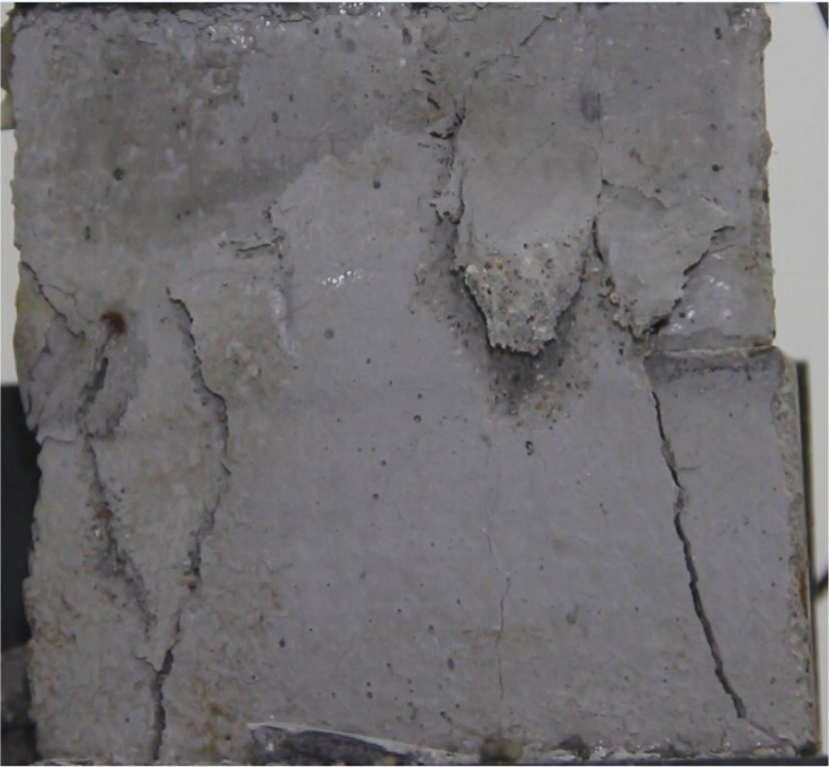
Photograph of a specimen showing surface spalling.

Figures 18, 19, 20, 21, 22 and 23 show photographs of a specimen with orientation 3 cracks rupture process under uniaxial compression.

As can be seen from Figs. 18, 19, 20, 21, 22 and 23. The first was tensile wing crack extending downwards from the tip of the fissure on the right side, followed by a tensile crack extending from the top face of the rock towards the fissure, followed by a tensile crack extending downwards from the tip of the left crack. As the axial load increased, tensile crack were produced from the top and bottom surface of the rock mass. After that surface spalling was produced in the middle of the rock mass.

**Figure 24:**
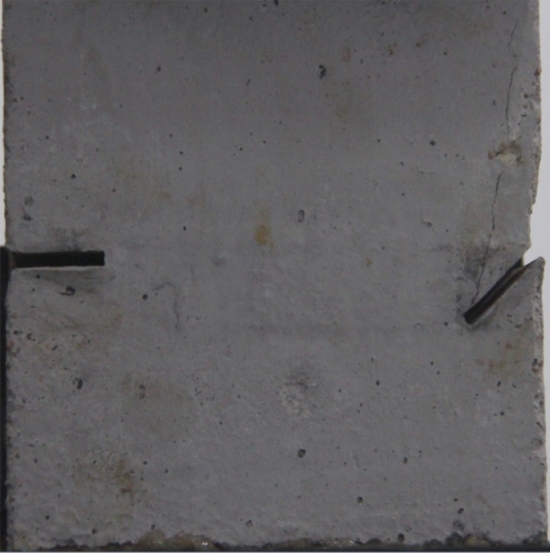
Photograph of a specimen showing anti-tensile wing crack appearing.

**Figure 25:**
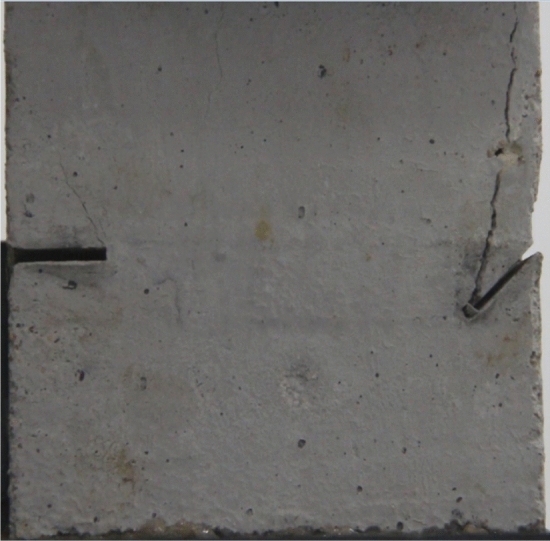
Photograph of a specimen showing anti-tensile wing crack Propagating and another anti-tensile wing crack appearing.

**Figure 26:**
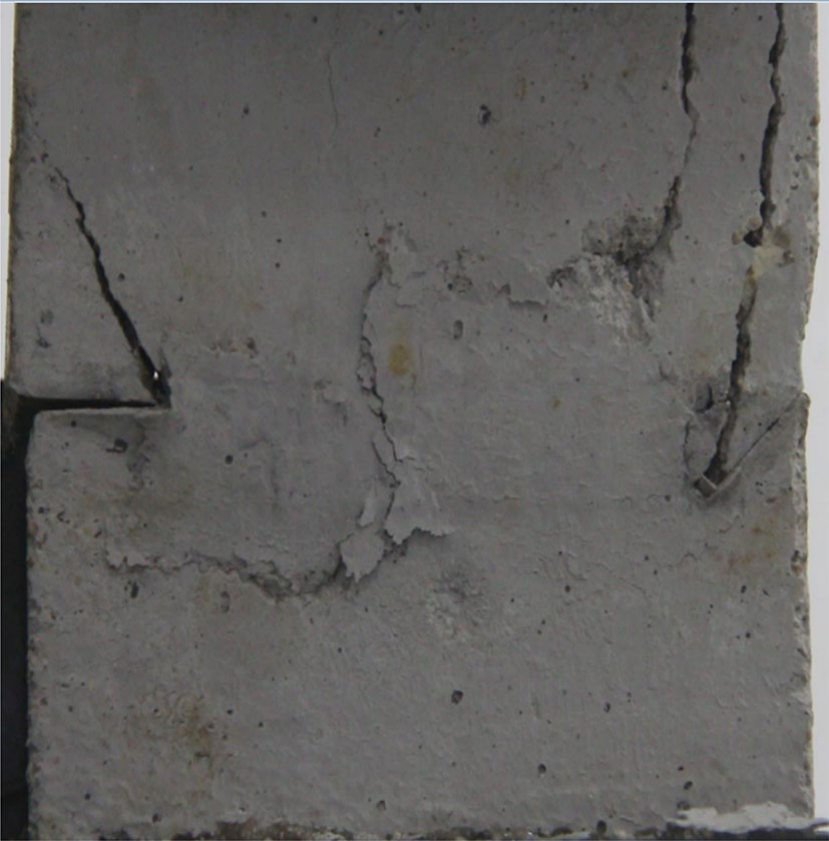
Photograph of a specimen showing tensile crack from the top surface caused by compression and transverse shear crack appearing.

**Figure 27:**
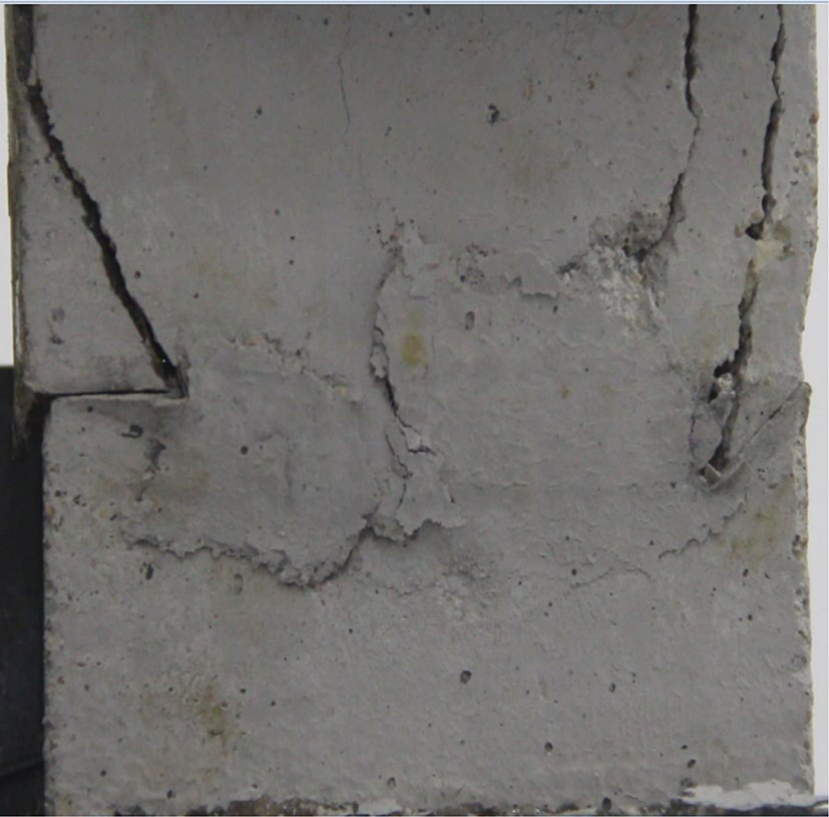
Photograph of a specimen showing transverse shear crack propagation.

Figures 24, 25, 26 and 27 show photographs of a specimen with orientation 4 cracks rupture process under uniaxial compression.

As can be seen from Figs. 24, 25, 26 and 27. The first was a anti-tensile wing crack extending upwards from the tip of the fissure on the right side of the rock mass, followed by a tensile crack extending towards the left boundary from the tip of the fissure on the left. After that a tensile crack extending downwards from the top of the rock mass was produced. Finally a transverse shear crack in the middle of the rock mass was produced.

### Type of crack

**Figure 28:**
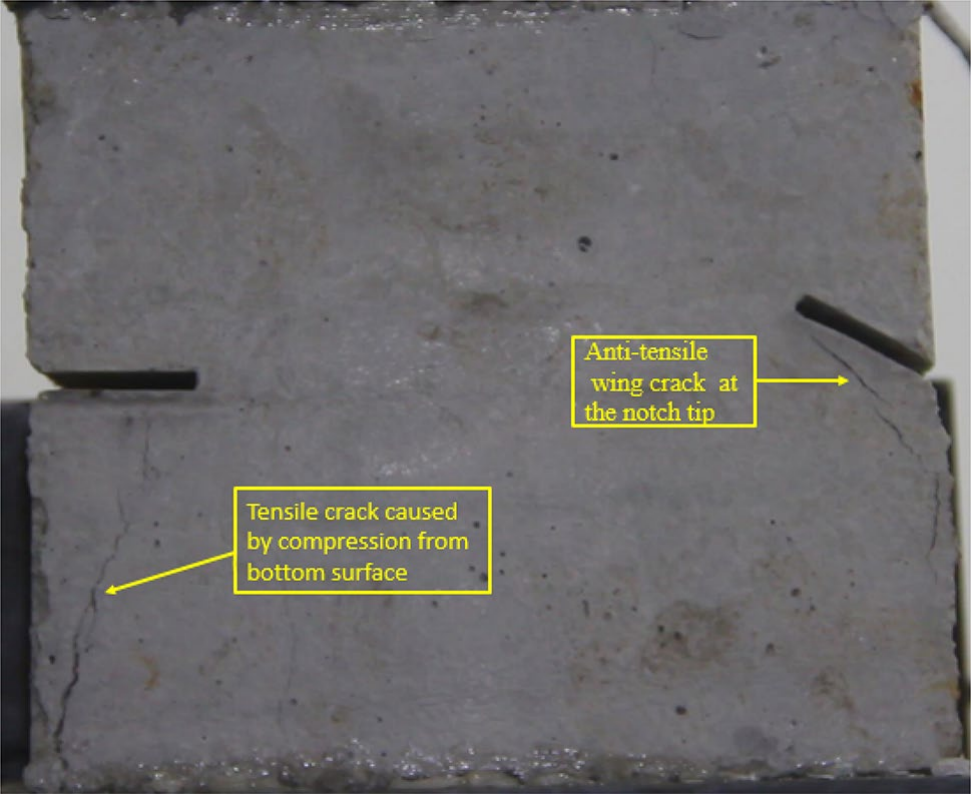
Photograph of a specimen showing tensile and anti-tensile crack.

**Figure 29:**
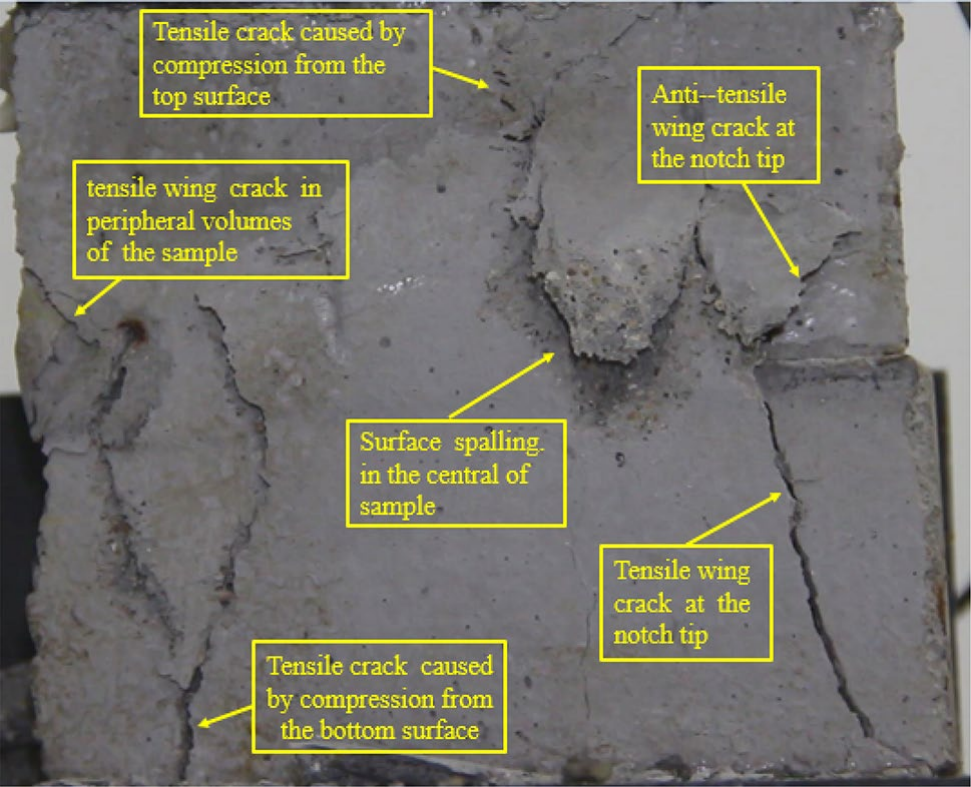
Photograph showing three types of tensile crack, two types of anti-tensile wing crack and surface spalling.

**Figure 30:**
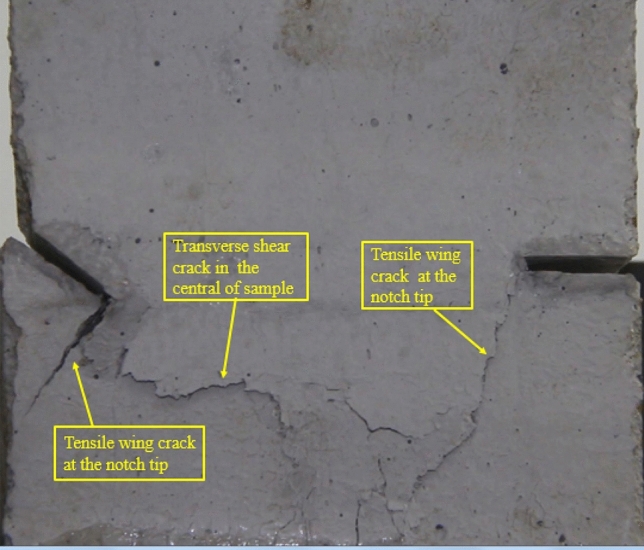
Photograph showing two tensile wing crack and a transverse shear crack.

There were six types of crack. They are shown in Figs. 28, 29 and 30 and listed below.

- Tensile wing crack (at the notch tip)
- Anti-tensile wing crack (at the notch tip)
- Tensile wing crack (in the peripheral volumes of the sample)
- Transverse shear crack (in the central of sample)
- Compression induced tensile crack (Tensile crack caused by compression from the top or the bottom surface)
- Surface spalling (in the central of sample)

### Crack length and their influence on fracturing

**Figure 31:**
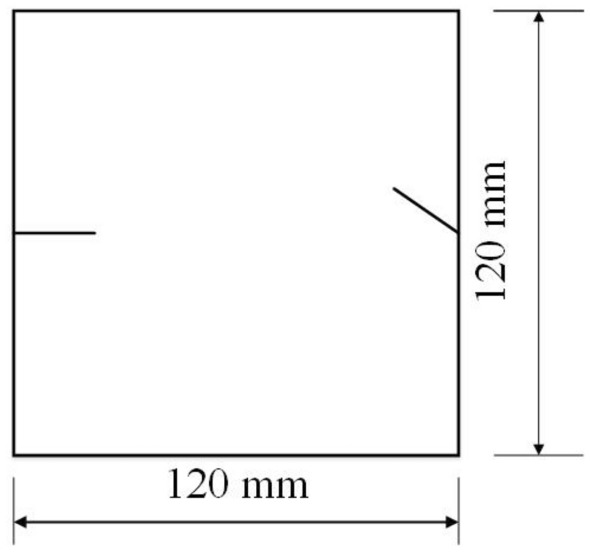
Specimen with two 20 mm long cracks.

**Figure 32:**
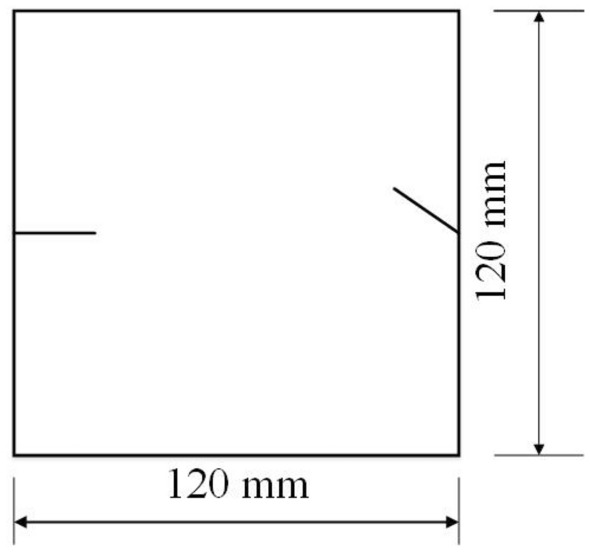
Specimen with two 30 mm long cracks.

To study the effects of different crack lengths on the crack patterns, two specimens with identical crack orientations but different crack lengths (20 mm and 30 mm) were prepared (Figs. 31, 32).

**Figure 33:**
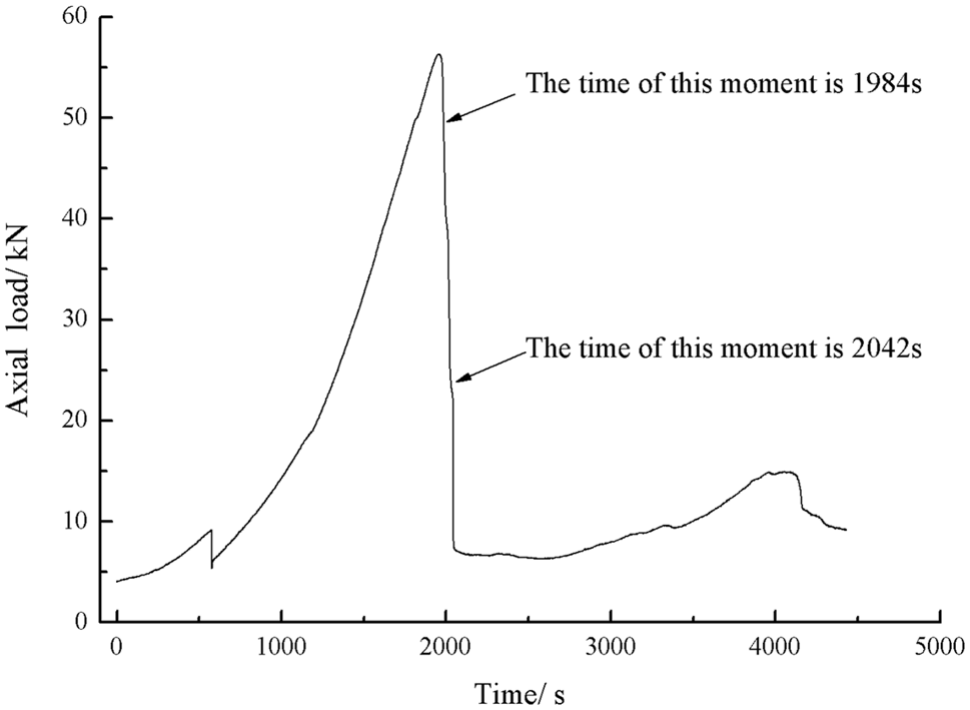
Axial load–time curve for a specimen with 20 mm long cracks. Photographs of this specimen taken at the times marked on the curve are shown as Figs. 34 and 35.

**Figure 34:**
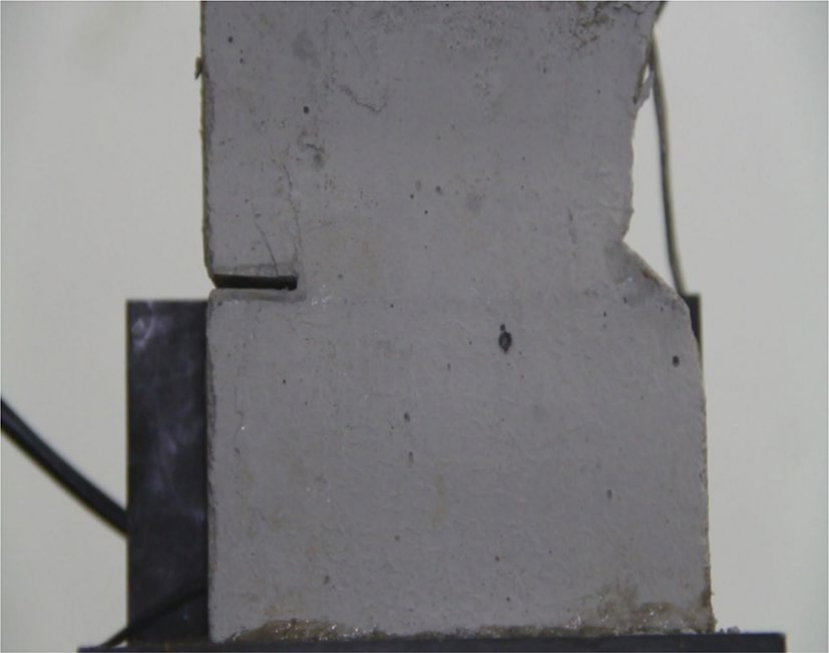
Photograph of a specimen with 20 mm long cracks under uniaxial compression at *t* = 1984s showing the crack developed at that time.

**Figure 35:**
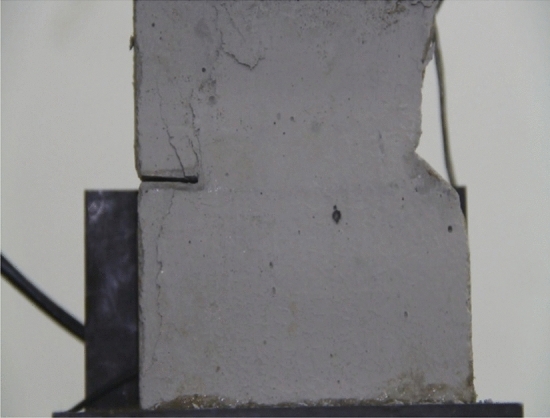
The same specimen shown in Fig. 33 at *t* = 2042s.

Figure 33 shows the Axial load-Time curve for a specimen with 20 mm long cracks. Figures 34 and 35 are photographs showing the crack in that specimen after 1984s and 2042s of compression, respectively.

In the specimen with 20 mm long cracks, the stress decreased from its peak at 1984s after the experiment was begun. At this time, the upper right side of the specimen was cut by a crack and the stress decreased abruptly. By 2042s, crack had propagated upward and downward from the tip of the left side crack. Both the specimen’s strength and the stress were greatly reduced.

**Figure 36:**
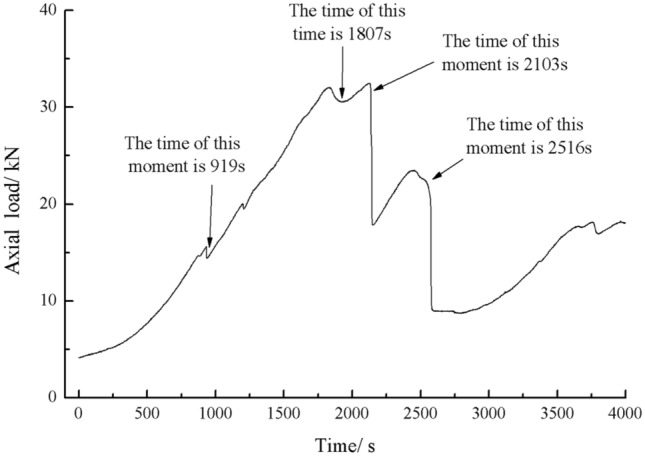
Axial load–Time curve for a specimen with 30 mm long cracks. Photographs of this specimen taken at the times marked on the curve are shown as Figs. 37, 38, 39 and 40.

**Figure 37:**
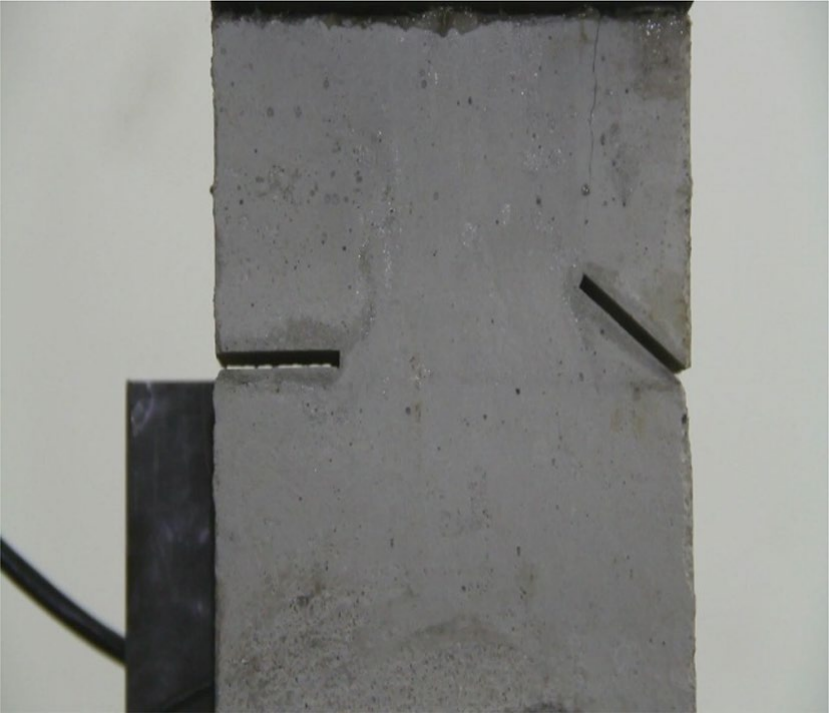
Photograph of a specimen with 30 mm long cracks under uniaxial compression at *t* = 919 s showing the crack developed at that time.

**Figure 38:**
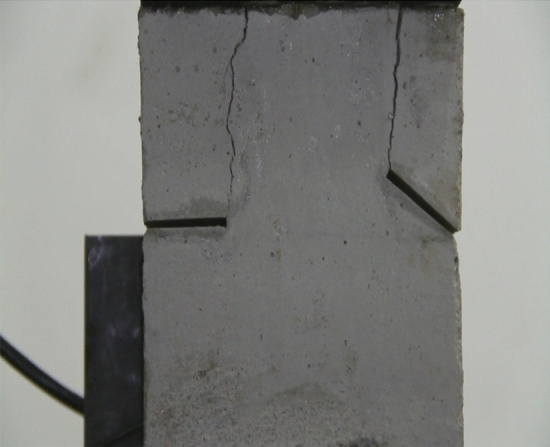
The same specimen shown in Fig. 36 at *t* = 1807s.

**Figure 39:**
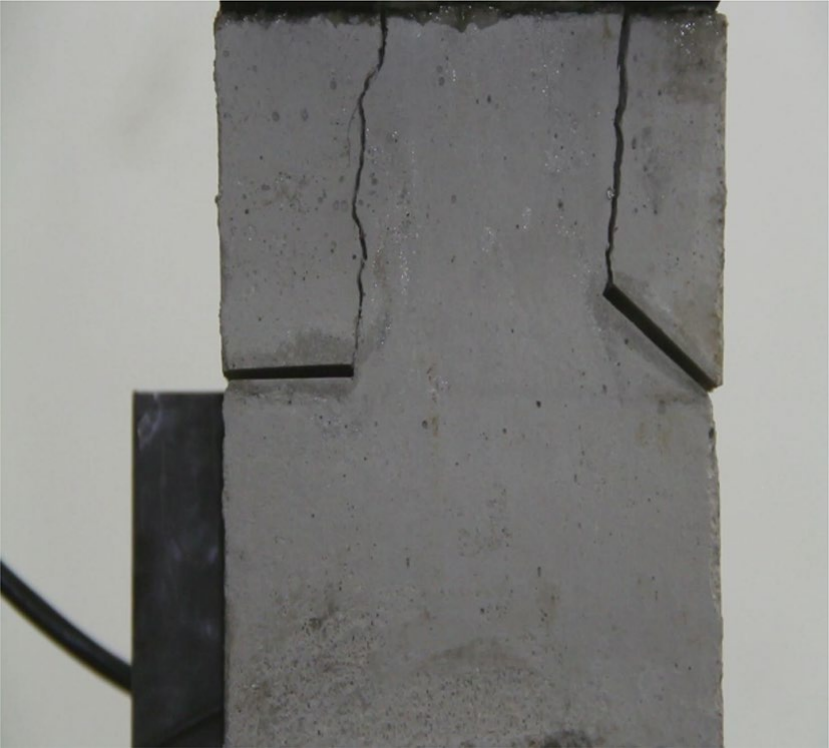
The same specimen shown in Fig. 36 at *t* = 2103 s.

**Figure 40:**
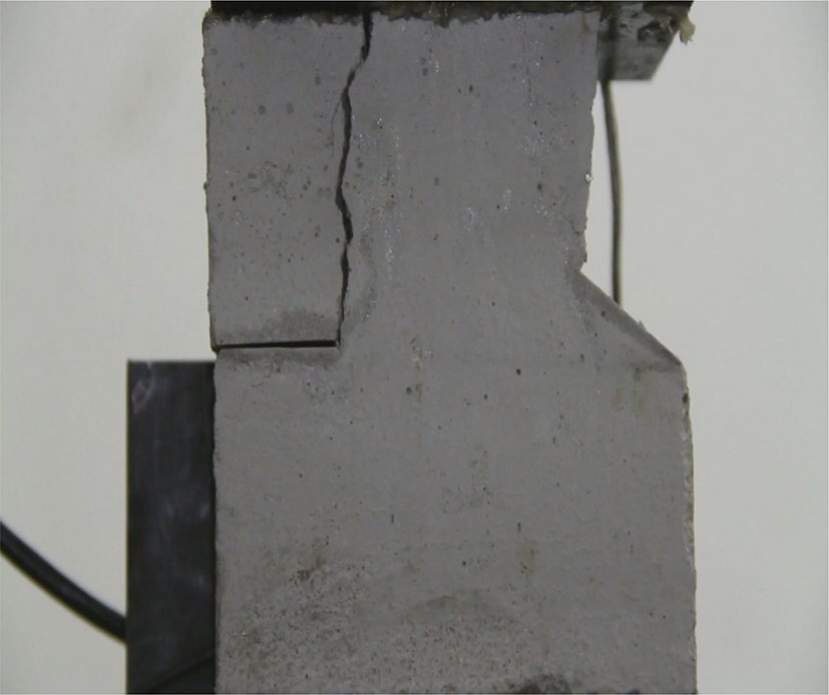
The same specimen shown in Fig. 36 at *t* = 2561 s.

Figure 36 shows the Axial load-Time curve for a specimen with 30 mm long cracks. Figures 37, 38, 39 and 40 are photographs showing the crack in that specimen after 919 s, 1807s, 2103 s, and 2561 s of compression, respectively.

The rock mass with 2 cm long fissures soon developed cracks from the tip of the fissures on the left side. The cracks extended towards the top and bottom surface, which in turn formed cracks through the top and bottom.

The rock mass with 3 cm long fissures only developed cracks from the tip of the fissure to the top surface firstly and did not develop cracks from the tip of the fissure to the bottom surface. The rock is not quickly damaged. The rock is broken gradually.

**Figure 41:**
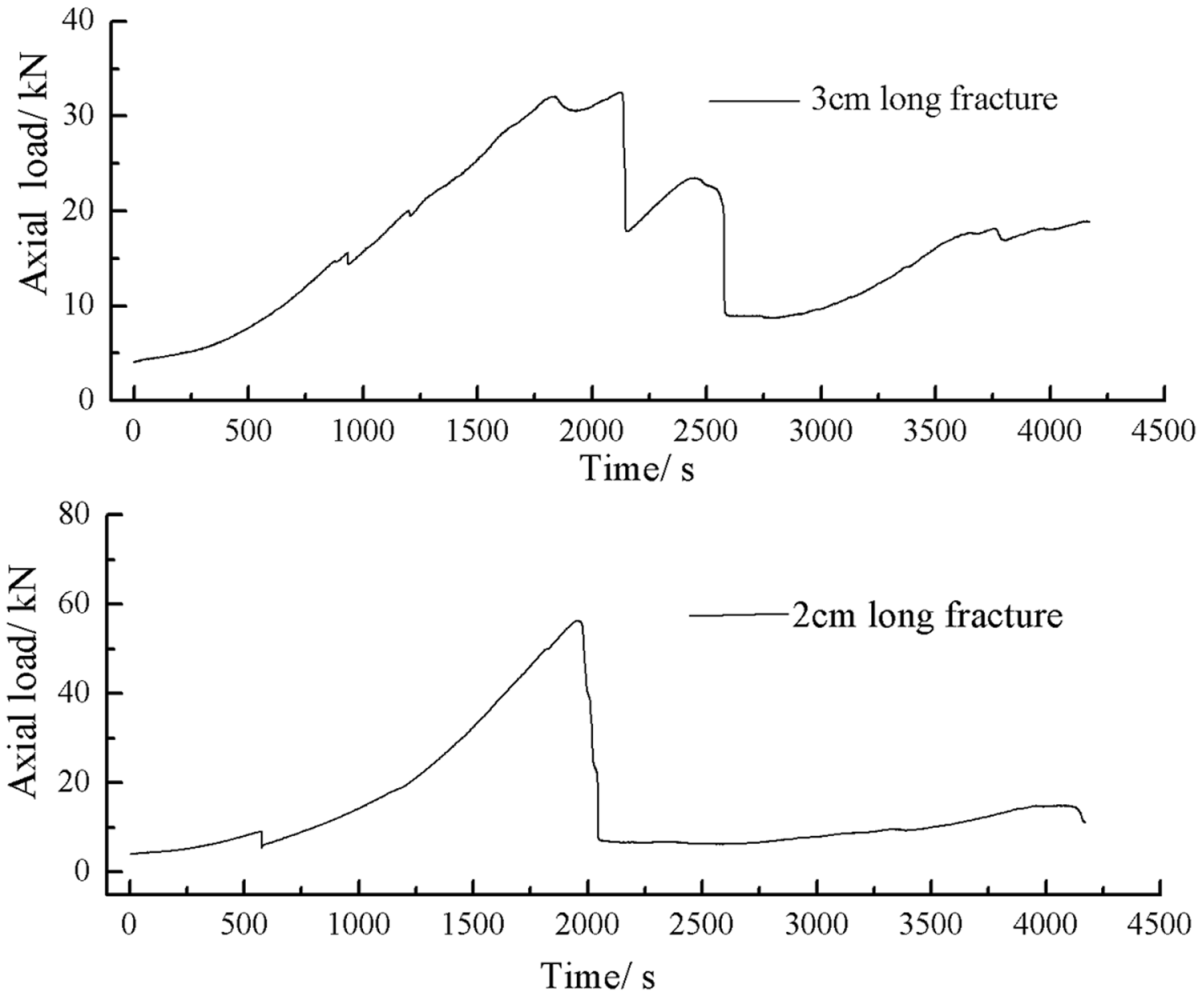
Axial load-Time curves for rock masses with two fracture lengths.

The axial load time curves for two crack lengths of rock are shown in the Fig. 41.

As can be seen from Fig. 41, the peak axial load was greater for a rock mass with 2 cm long crack. The shorter the crack length the less strength damage to the rock mass.

The rock mass with 2 cm long crack was brittle. The rock mass with 2 cm long cracks soon developed cracks from the tip of the fissures on the left side. The cracks extended towards the top and bottom surface, which in turn formed cracks through the top and bottom. The rock mass lost its strength quickly.

The cracks were longer and the damage to the strength of the rock was greater. The rock mass with 3 cm long crack only developed cracks from the tip of the fissure to the top surface firstly and did not develop cracks from the tip of the fissure to the bottom surface. The rock is not quickly damaged. The rock is broken gradually. The strength of the rock mass is lost gradually with the expansion of the fissures.

### Crack length and their influence on acoustic emission

**Figure 42:**
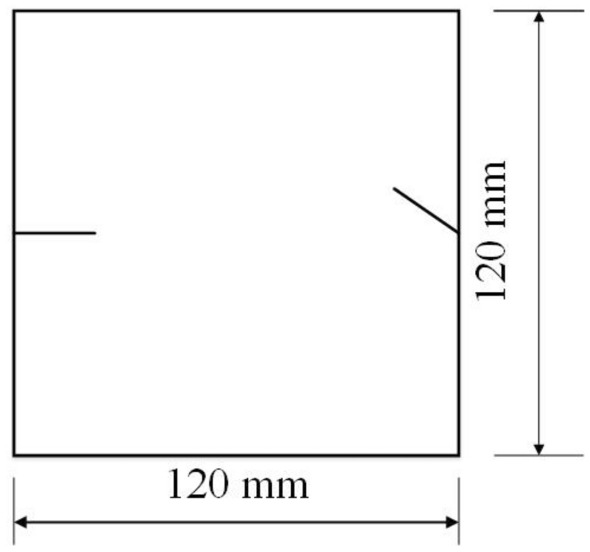
Specimen with two 20 mm long cracks.

**Figure 43:**
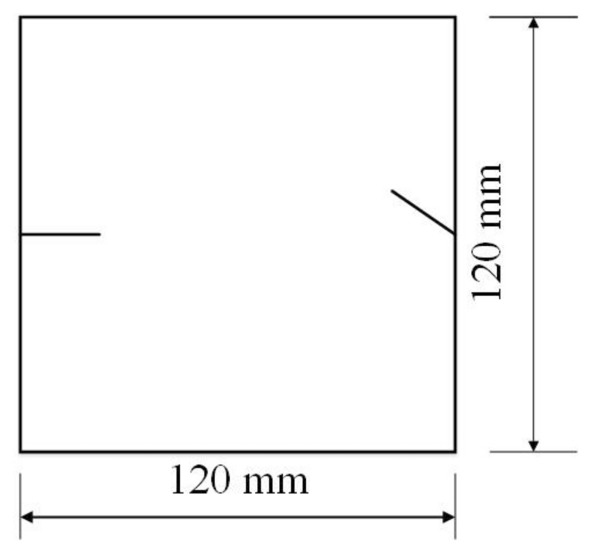
Specimen with two 30 mm long cracks.

To study the effects of different crack lengths on the acoustic emission, two specimens with identical crack orientations but different crack lengths (20 mm and 30 mm) were prepared (Figs. 42, 43).

The acoustic emission energy-axial load curves of the specimens with 2 cm, 3 cm crack as shown below.

**Figure 44:**
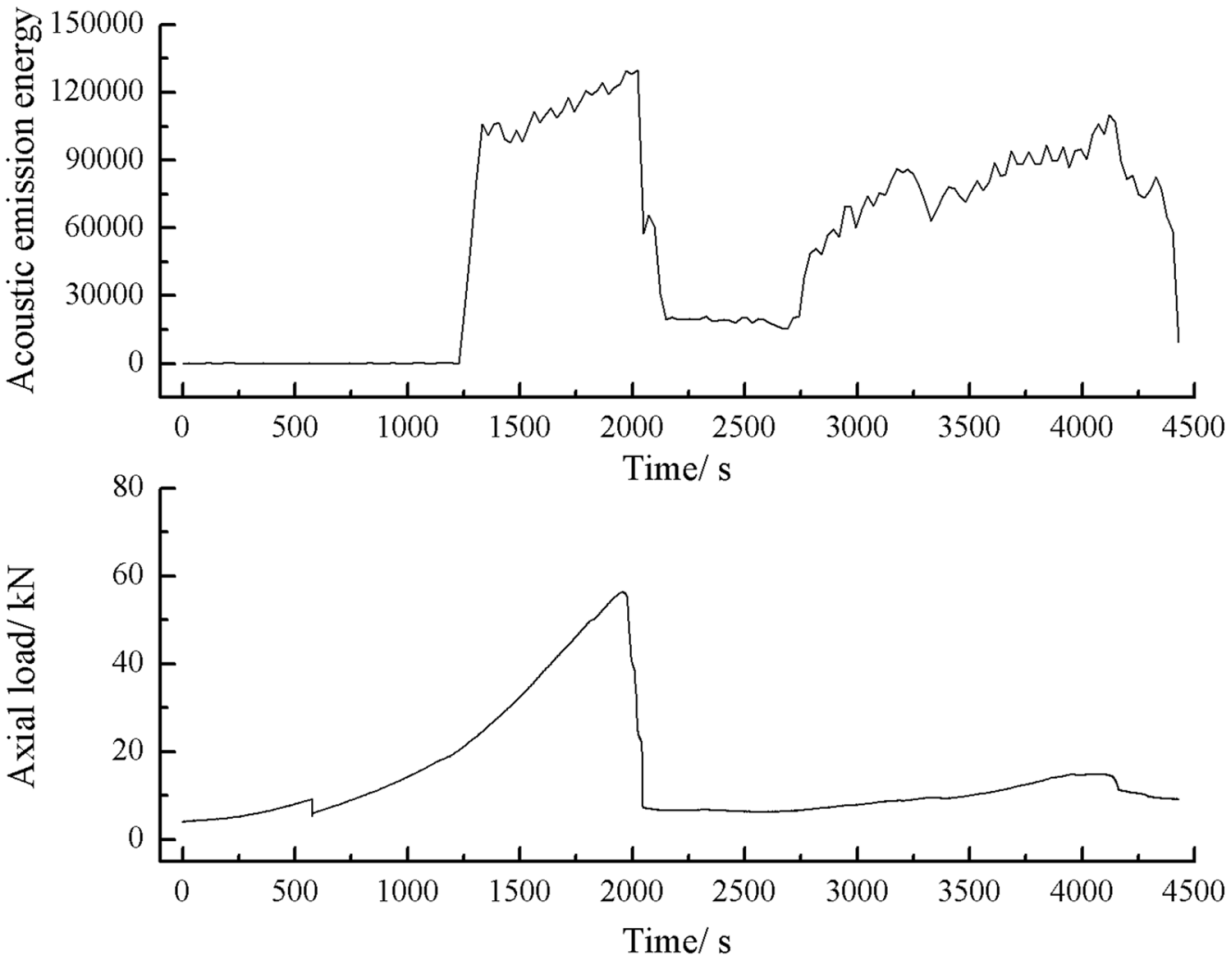
Acoustic emission energy-Axial load of the specimen with 2 cm crack.

**Figure 45:**
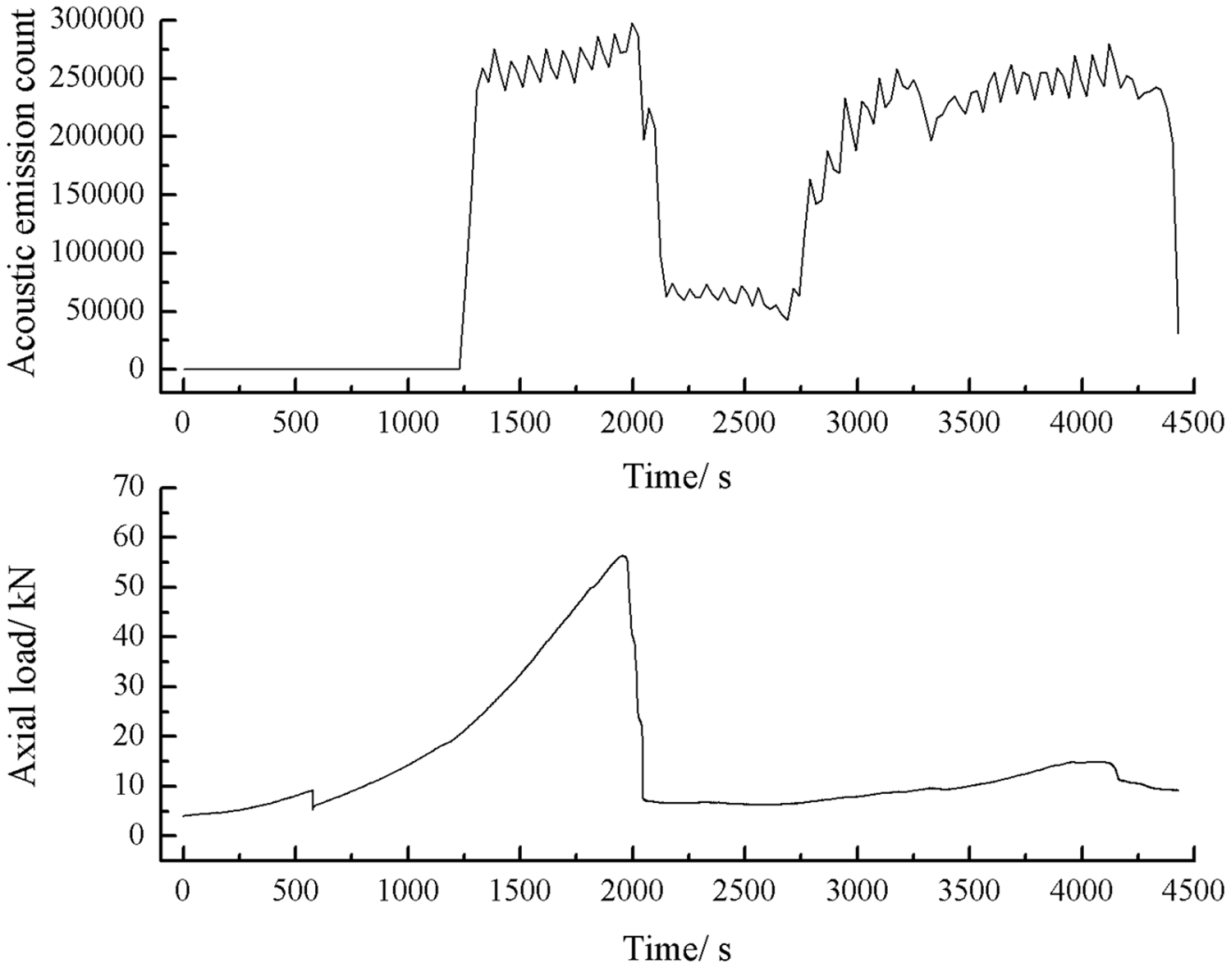
Acoustic emission count-Axial load of the specimen with 2 cm crack.

**Figure 46:**
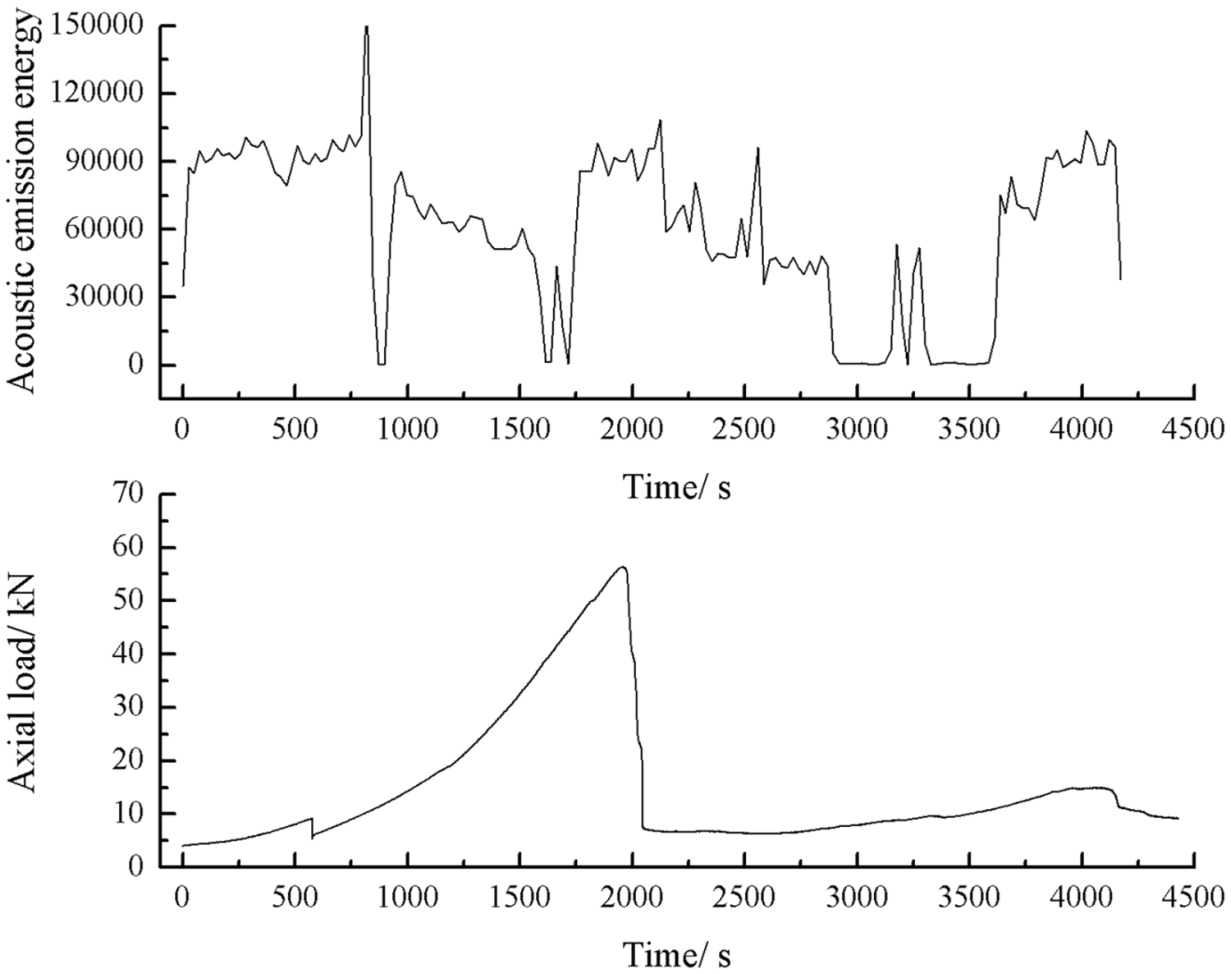
Acoustic emission energy-Axial load of the specimen with 3 cm crack.

**Figure 47:**
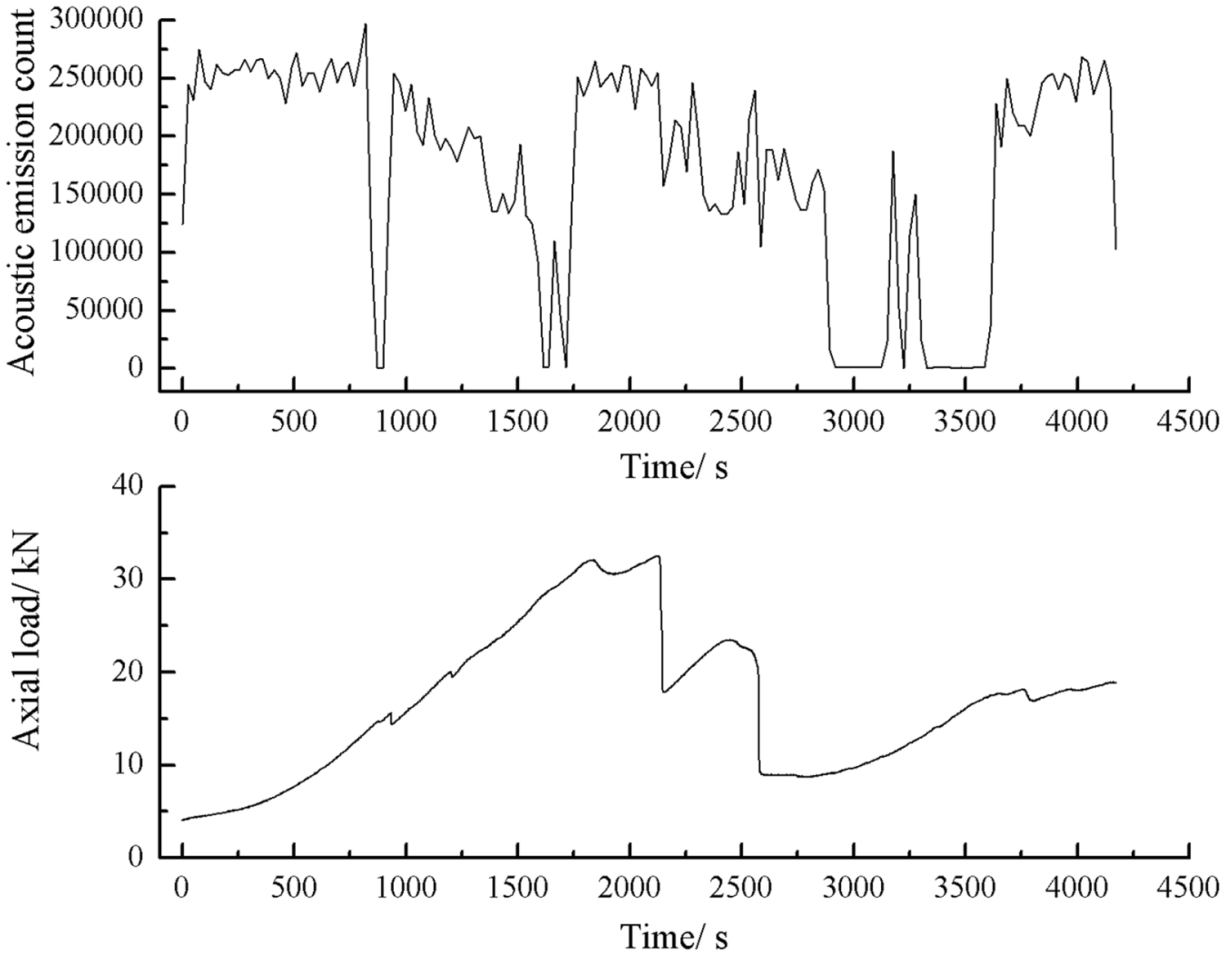
Acoustic emission count-Axial load of the specimen with 3 cm crack.

As can be seen from Figs. 44, 45, 46 and 47, there was a good correlation between the change in axial load and the change in acoustic emission energy of the rock mass. When the axial load was small, the acoustic emission energy was small. When the axial load increased, the acoustic emission energy also increased. When the axial load reached the peak, the acoustic emission energy also reached its maximum point. When the axial load dropped abruptly, the acoustic emission energy increased abruptly.

The rock mass with 2 cm long fissures soon developed cracks from the tip of the fissures on the left side. The cracks extended towards the top and bottom surface, which in turn formed cracks through the top and bottom. The acoustic emission rapidly reached the maximum point at brittle crack first.

The fracture initiation condition for the 2 cm long fracture was that an initial fracture extending from the right fracture tip to the top surface of the rock was produced at 1984s. The axial load dropped from 56 to 49.3 kn, corresponding to a high point in the acoustic emission energy and acoustic emission count diagram at 1984s.

The fissures were longer and the damage to the strength of the rock was greater. The rock mass with 3 cm long fissures only developed cracks from the tip of the fissure to the top surface firstly and did not develop cracks from the tip of the fissure to the bottom surface. The rock is not quickly damaged. The rock is broken gradually. The acoustic emission had several sudden rises and several sudden declines.

The fracture initiation condition for the 3 cm long fracture was that an initial fracture extending from the top of the rock towards the right-hand fracture tip was produced at 919 s. The axial load dropped from 15.6kn to 14.4kn, corresponding to a high point in the acoustic emission energy and the acoustic emission count diagram at 919 s.

### Particle size effects on fracturing and sample mechanical properties

**Figure 48:**
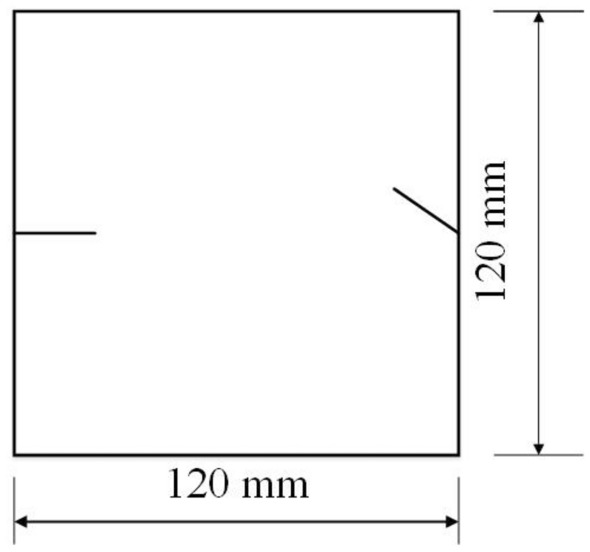
Both cracks 20 mm long, in specimens with different particle sizes.

To study the effects of particle size on the specimens’ crack patterns and the specimens’ mechanical properties, specimens with the same style cracks but made from cement mortar mixes with different sand particle sizes were produced. One specimen contained sand from the 1–2.56 mm fraction, a second specimen contained equal parts of 0–0.6 mm and 1–2.56 mm sand, and the third specimen had sand from all three size fractions and therefore contained sand in the whole 0–2.56 mm particle size range. The orientations of the cracks in these three specimens are shown in Fig. 48.

#### Particle sizes and fracturing

1. Fracturing in a specimen produced from a cement mortar mix containing sand in the 1–2.56 mm particle size range.
2. Fracturing in a specimen produced from a cement mortar mix containing sand in the 0–0.6 mm and 1–2.56 mm particle size ranges.
3. Fracturing in a specimen produced from a cement mortar mix containing sand in the 0–2.56 mm particle size range.

**Figure 49:**
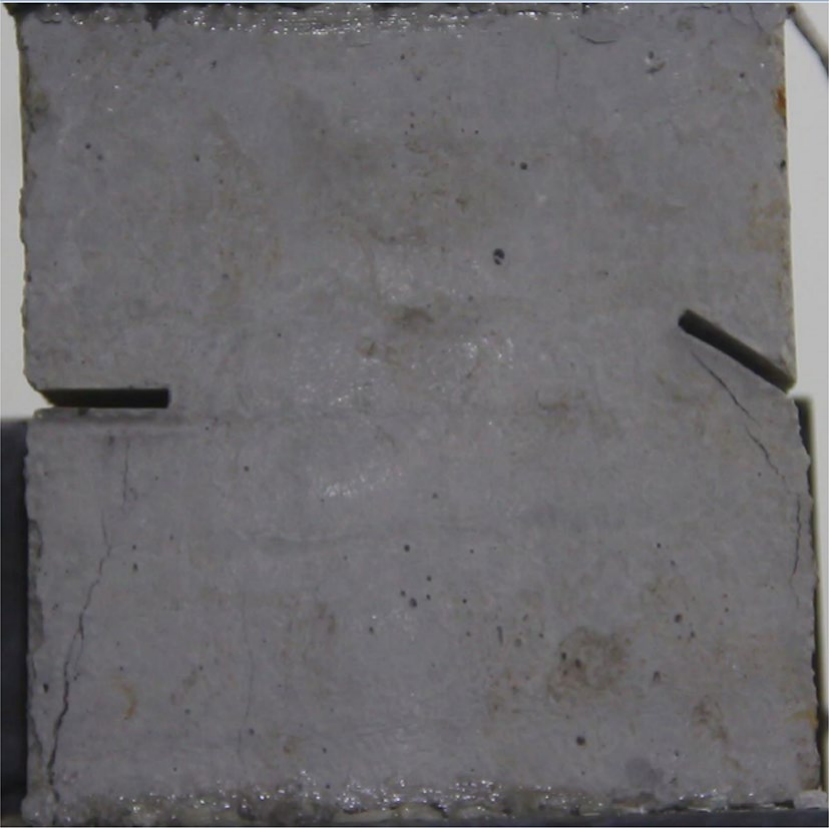
Photograph of a specimen showing tensile crack from the bottom surface caused by compression and anti-tensile wing crack appearing.

**Figure 50:**
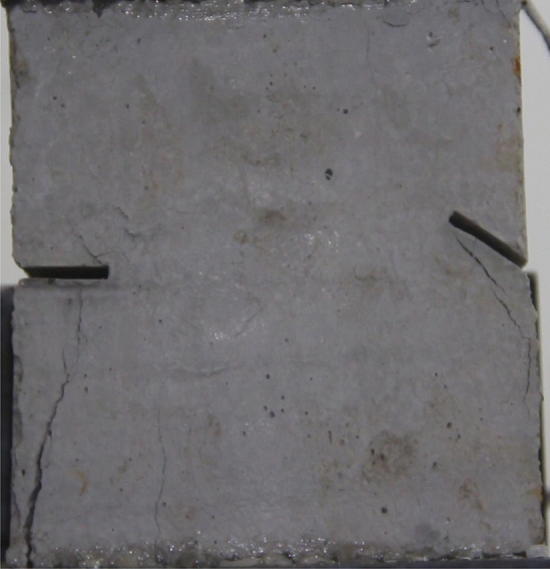
Photograph of a specimen showing tensile crack from the bottom surface caused by compression propagating.

**Figure 51:**
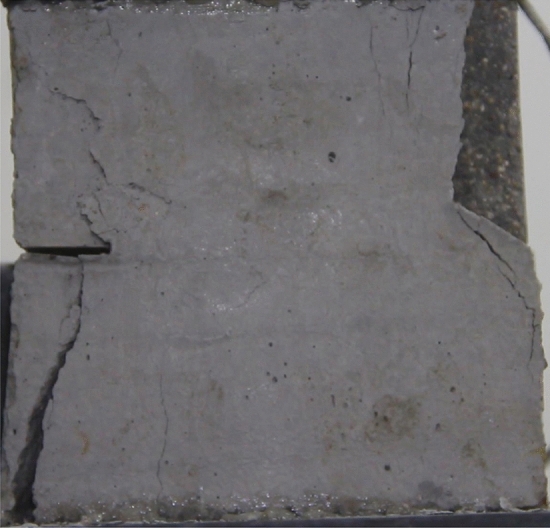
Photograph of a specimen showing tensile wing crack and tensile crack from the top surface caused by compression appearing.

**Figure 52:**
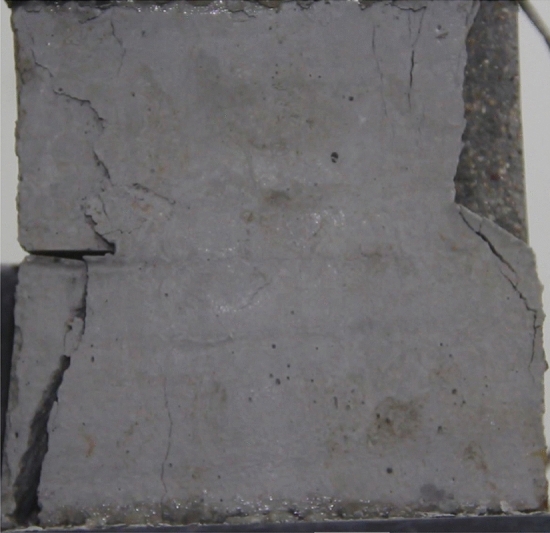
Photograph of a specimen showing tensile crack from the top surface caused by compression propagating.

**Figure 53:**
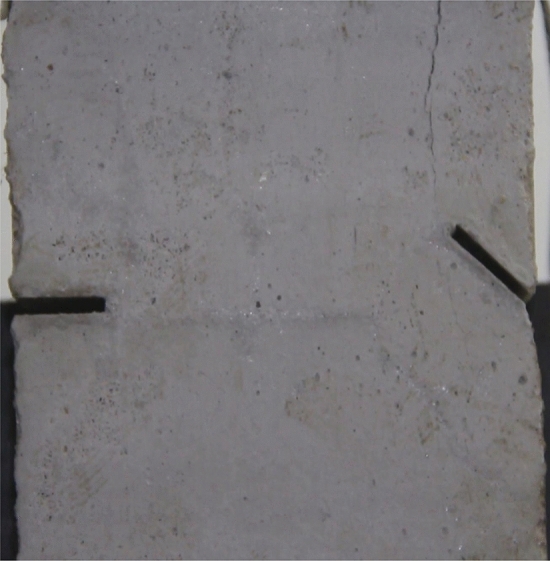
Photograph of a specimen showing tensile crack from the top surface caused by compression appearing.

**Figure 54:**
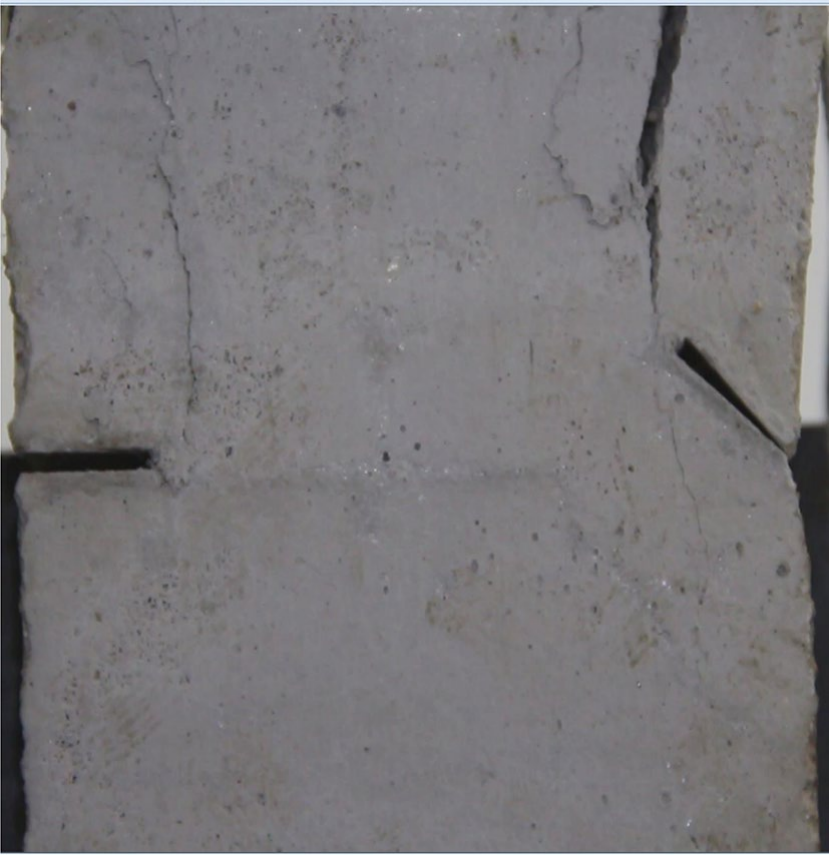
Photograph of a specimen showing tensile crack from the top surface caused by compression propagating.

**Figure 55:**
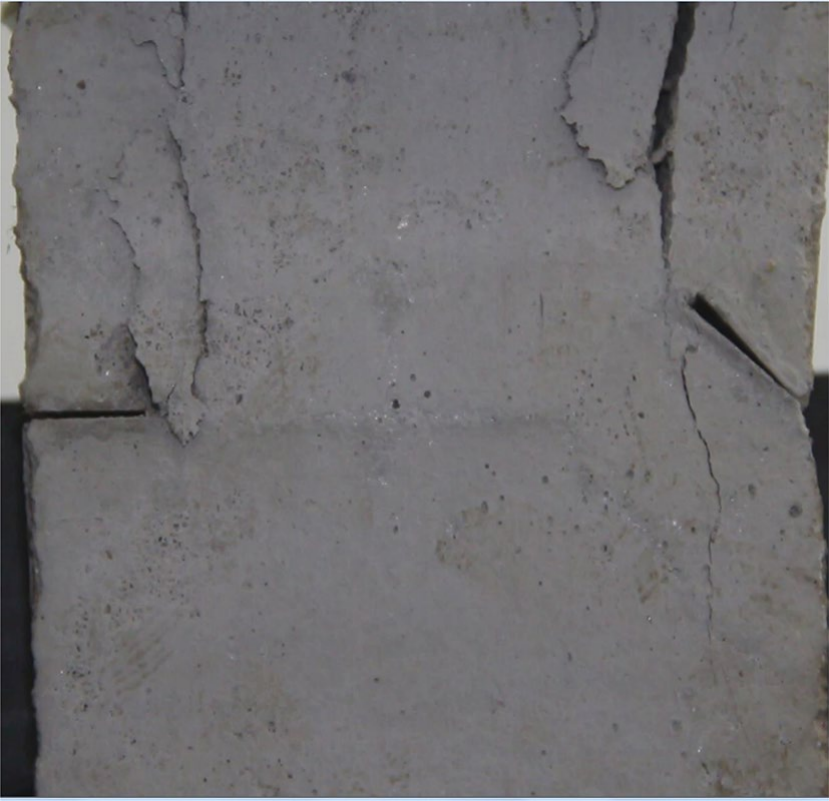
Photograph of a specimen showing anti-tensile wing crack.

**Figure 56:**
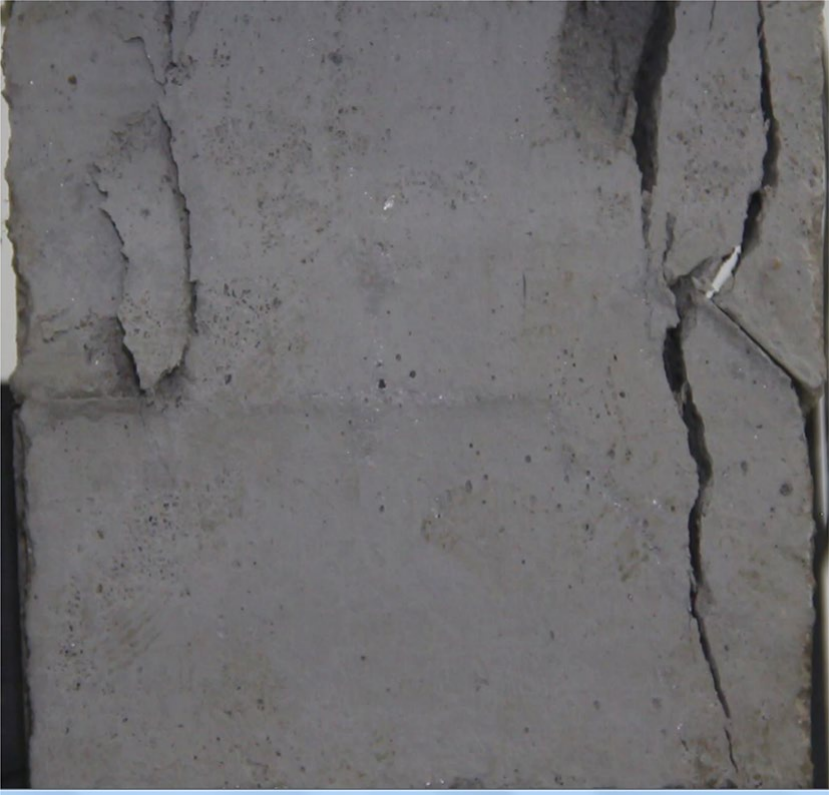
Photograph of a specimen showing anti-tensile wing crack propagating and crack penetration.

**Figure 57:**
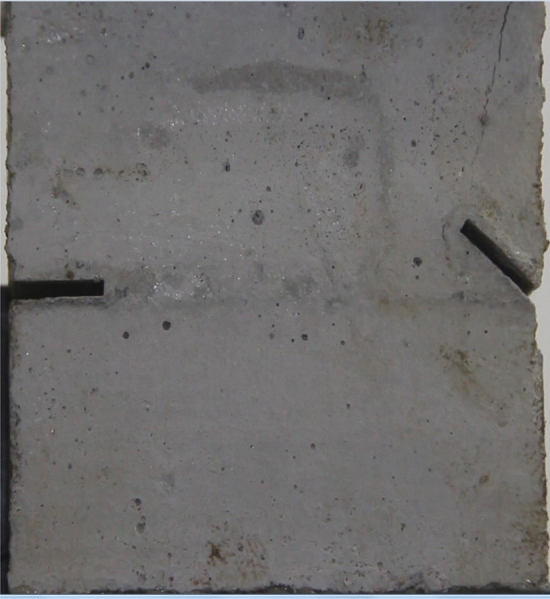
Photograph of a specimen showing tensile crack from the top surface caused by compression appearing.

**Figure 58:**
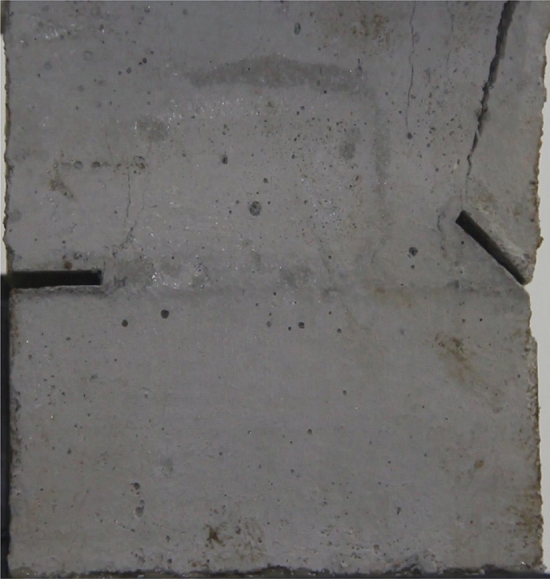
Photograph of a specimen showing tensile crack from the top surface caused by compression propagating.

**Figure 59:**
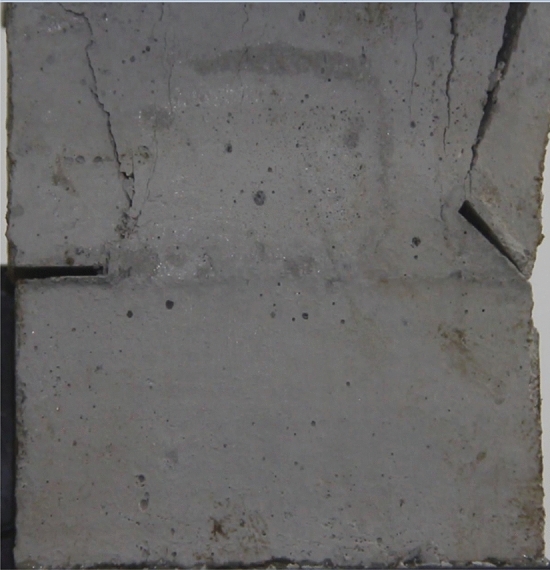
Photograph of a specimen showing tensile crack from the top surface caused by compression propagating.

**Figure 60:**
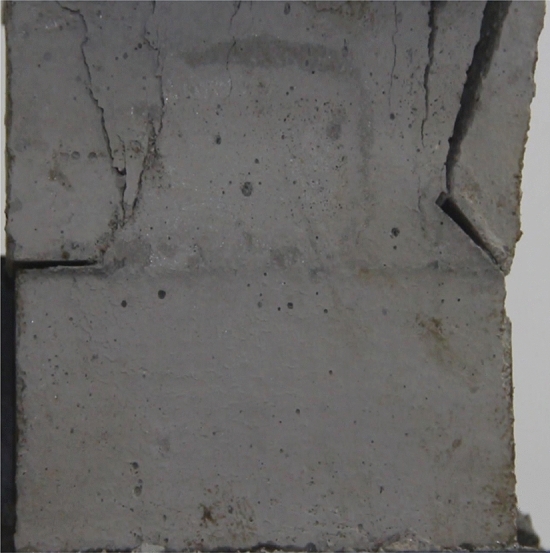
Photograph of a specimen showing tensile crack from the top surface caused by compression propagating.

Figures 49, 50, 51, 52, 53, 54, 55, 56, 57, 58, 59 and 60 show that fracturing specimens comprising various grain sizes first produced wing cracks that extended from the bottom of the specimen to the tip of the left crack. Other crack also started laterally and propagated towards the tip of the crack on the right side. They are anti-tensile fissures. Specimens with two ranges of grain sizes or a wide range of grain sizes produced stretching cracks that extended downward from the top of the fissure when they are compressed. At the same time, wing cracks that extended upward were produced, followed by anti-tensile cracks that extended downward. These cracks were initiated at different points and propagated in different directions.

#### Particle sizes and mechanical properties

**Figure 61:**
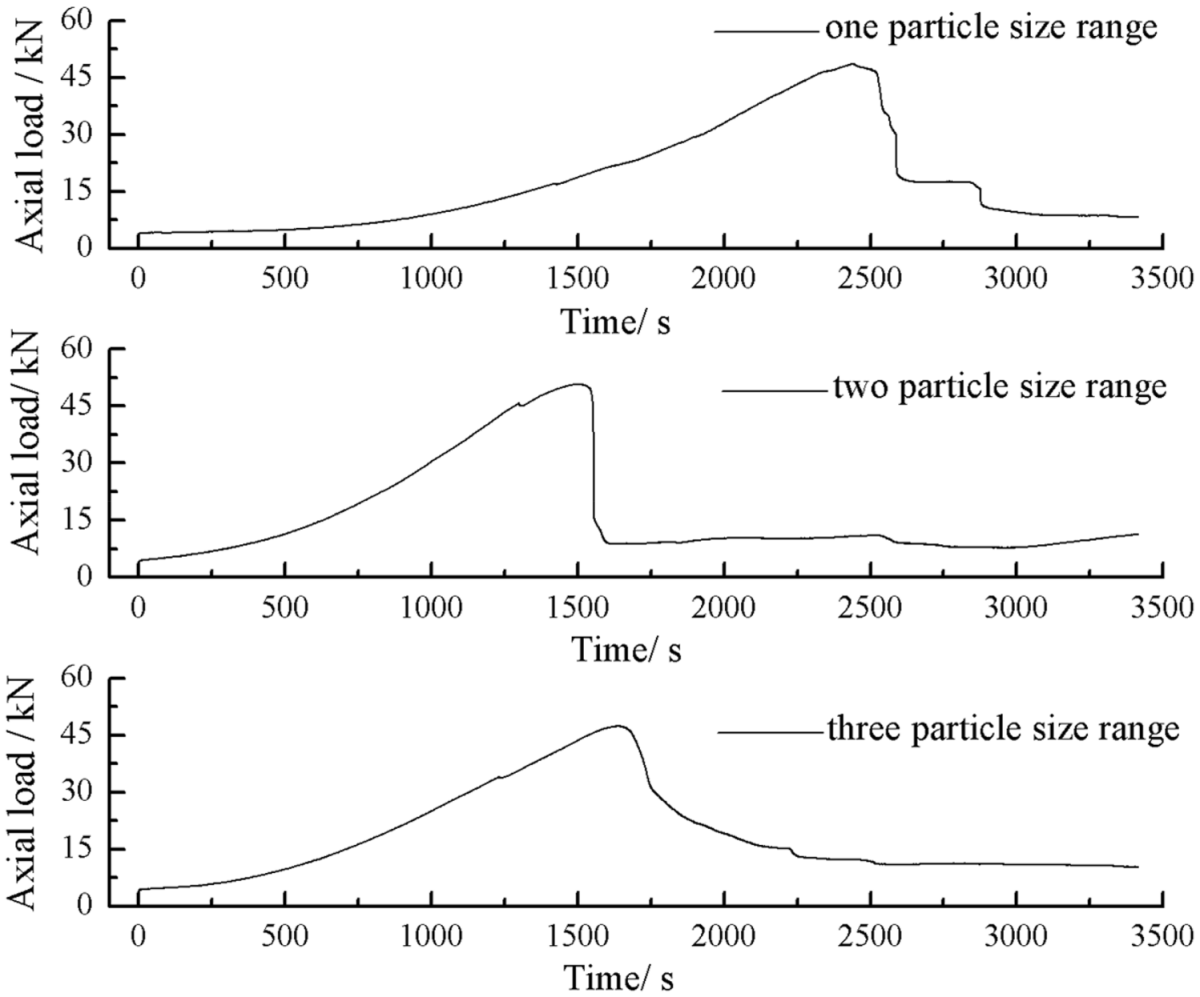
Axial load–Time curves for specimens containing three types of particle size ranges.

**Figure 62:**
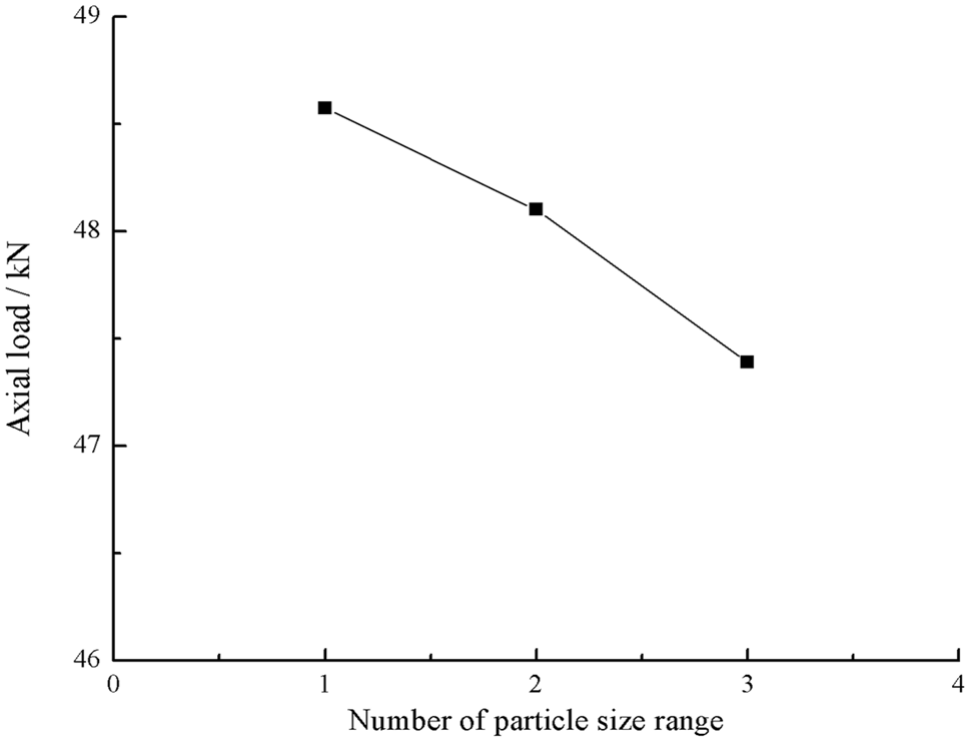
Graph of peak Axial load vs. Number of particle size ranges.

As can be seen from Figs. 61 and 62, the rock masses comprised of one and three-grain size ranges were progressively fractured. The axial load decreased gradually with time. The rock masse comprised of two-grain size ranges were brittle fractures. The axial load decreased rapidly after it reached its peak value.

The peak axial load for the specimen comprised of one grain size range was the largest. The peak axial load for the specimen comprised of three grain size ranges was the smallest. The specimen comprised of one grain size range had the largest average grain size.

### Particle size effects on acoustic emission

**Figure 63:**
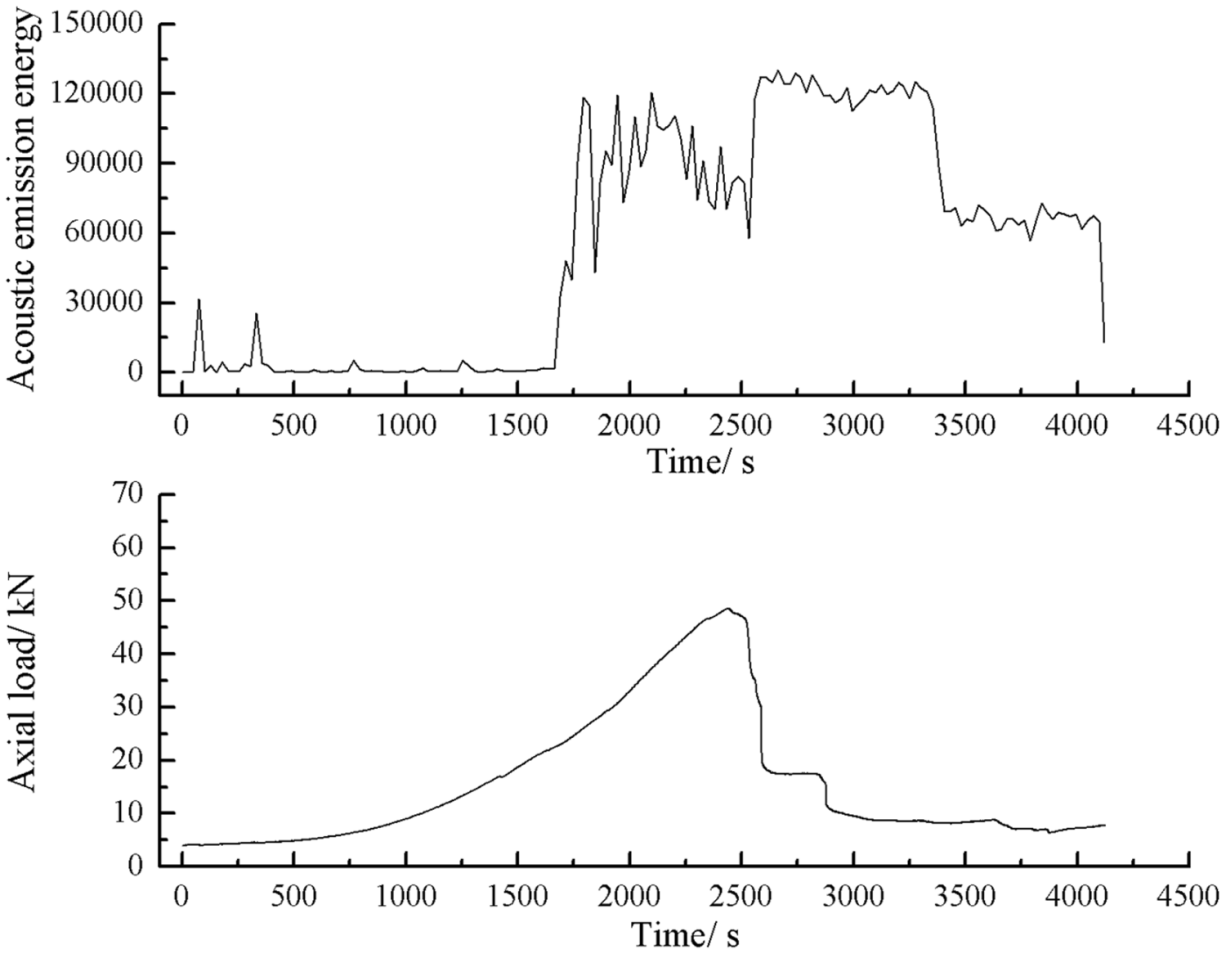
Acoustic emission energy-Axial load curve of the rock mass comprised of one grain size range.

**Figure 64:**
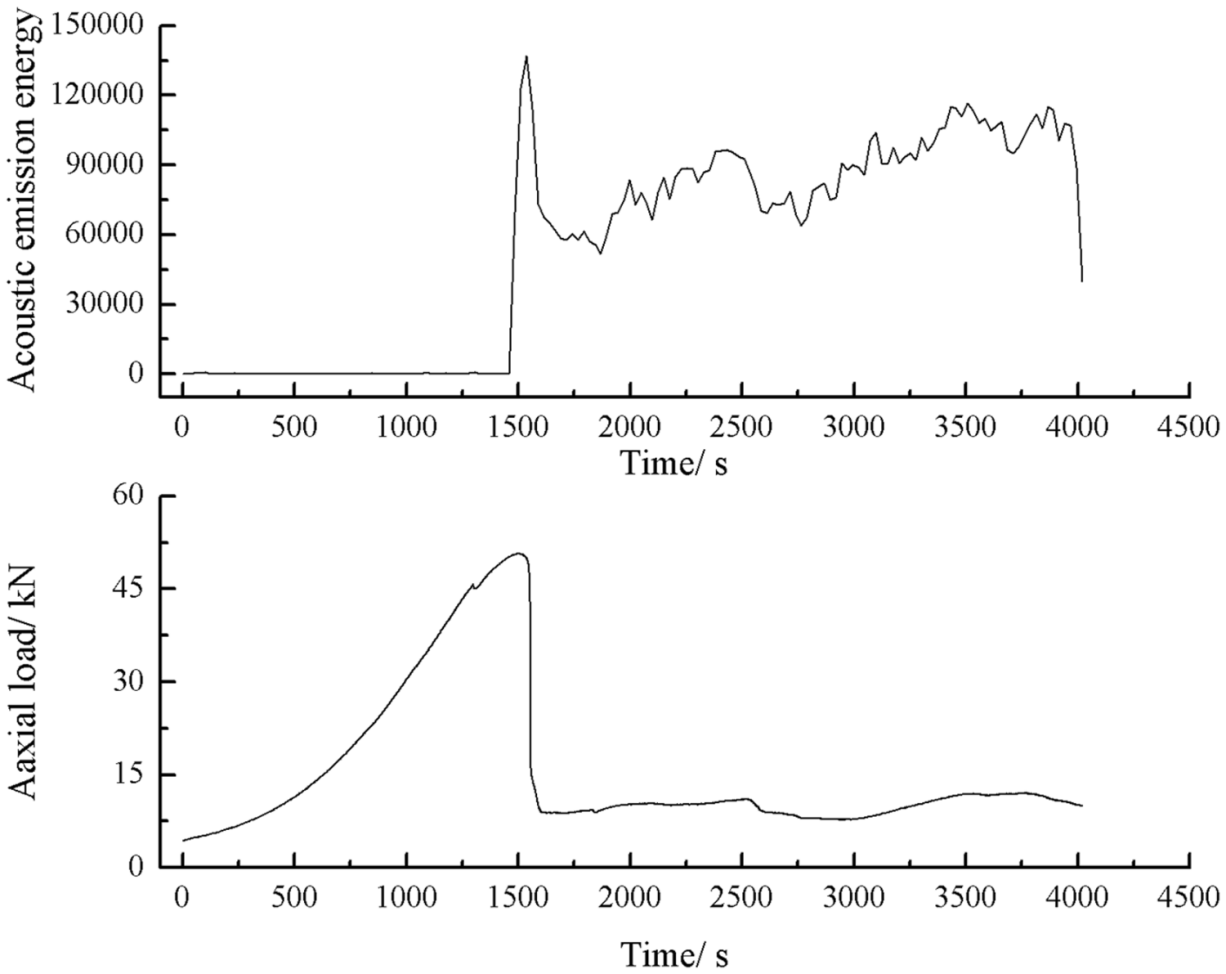
Acoustic emission energy-Axial load curve of the rock mass comprised of two grain size ranges.

**Figure 65:**
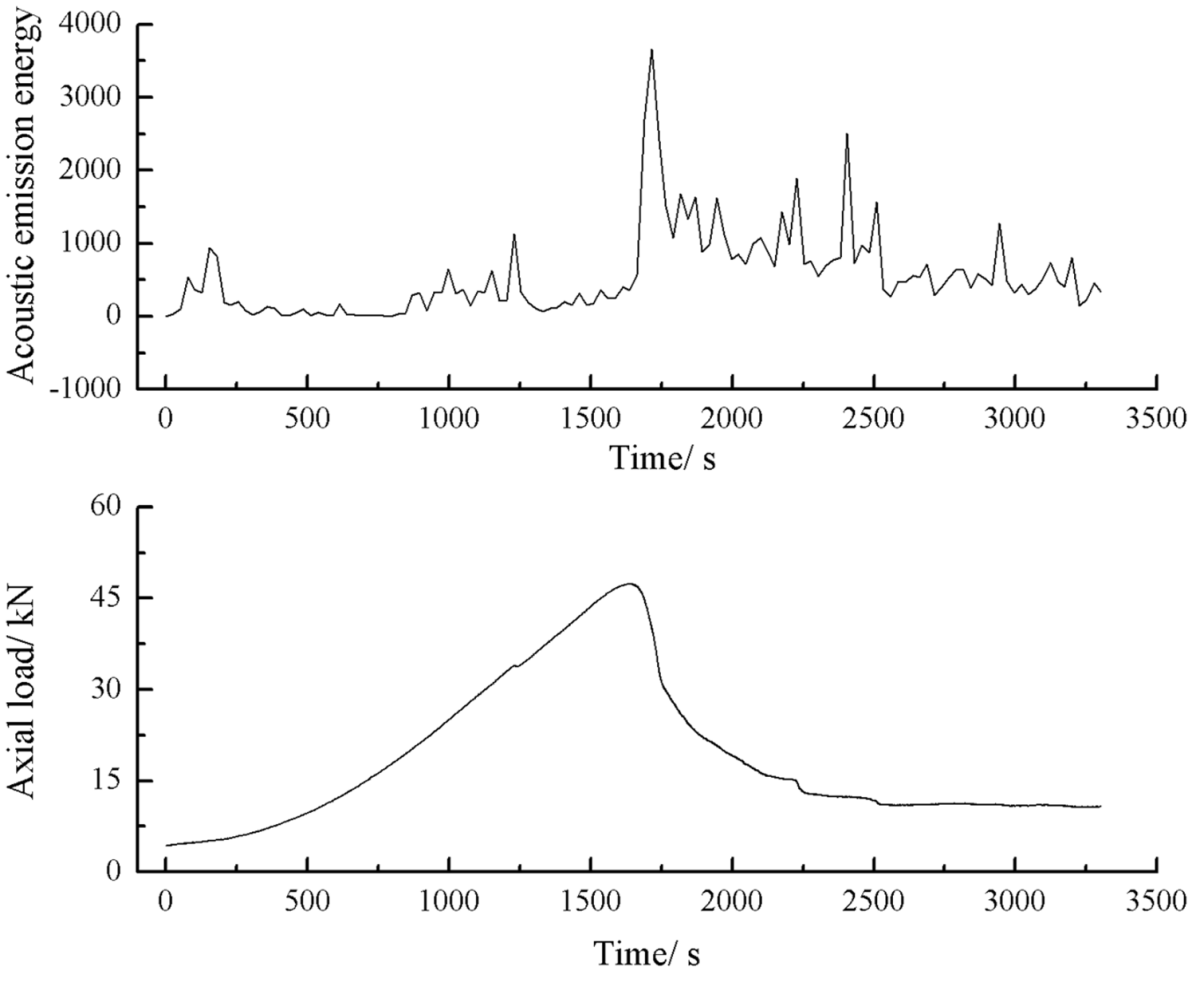
Acoustic emission energy-Axial load curve of the rock mass comprised of three grain size ranges.

As can be seen from Figs. 63, 64 and 65, there was a good correlation between the change in axial load and the change in acoustic emission energy of the rock mass. When the axial load was small, the acoustic emission energy was small. When the axial load dropped abruptly, the acoustic emission energy increased abruptly.

The rock masses comprised of one and three grain size ranges were broken gradually. Their Acoustic emission rose and fell rapidly with the gradual crack of the rock mass several times. The rock mass comprised of two grain size ranges was brittle crack, and the acoustic emission was strongest when the rock mass was penetrated and destroyed.

## Conclusions

This paper was a preliminary study, focusing on the fracture mode, fracture pattern and type of fracture produced. The next step will be to focus on the fracture initiation conditions, fracture conditions and fracture processes, taking into account the strength factor, fracture toughness, energy release rate and energy conservation of the rock mass.

This paper studied the influence of fissure patterns on the crack patterns and process of the rock mass. This paper studied the influence of fissure length and grain size composition on the crack characteristics, mechanical characteristics, and acoustic emission characteristics of the rock mass. The conclusions are as follows.

1. Under uniaxial compression, tensile wing crack, anti-tensile wing crack, transverse shear crack, compression induced tensile crack, and surface spalling are generated in specimens with different crack orientations.
2. The rock mass with 2 cm long fissures soon developed cracks from the tip of the fissures on the left side. The cracks extended towards the top and bottom surface, which in turn formed cracks through the top and bottom. The rock mass lost its strength quickly. The acoustic emission rapidly reached the maximum point at brittle crack first.
3. The rock mass with 3 cm long fissures only developed cracks from the tip of the fissure to the top surface firstly and did not develop cracks from the tip of the fissure to the bottom surface. The rock is not quickly damaged. The rock is broken gradually. The strength of the rock mass is lost gradually with the expansion of the fissures. The acoustic emission had several sudden rises and several sudden declines.
4. The specimen comprised of one-grain size range generates wing crack that extends from the bottom of it to the tip of the left crack when the specimen is compressed. There are also lateral crack that extend to the tip of the crack on the specimen’s right side. These are anti-wing cracks. Crack in specimens comprised of two or three grain size ranges are tensile cracks that extend downward from the top of the specimen. Wing cracks that extend upward and anti-wing cracks that extend downward are also produced. The locations and directions of the crack produced in specimens comprised of one grain size range are different from the crack produced in specimens comprised of two and three-grain size ranges.
5. The rock masses comprised of one and three grain size ranges were progressively fractured. The axial load decreased gradually with time. The rock masse comprised of two grain size ranges were brittle fractures. The axial load decreased rapidly after it reached its peak value. The peak axial load for the specimen comprised of one grain size range was the largest. The peak axial load for the specimen comprised of three grain size ranges was the smallest. The specimen comprised of one-grain size range had the largest average grain size.
6. The rock masses comprised of one and three grain size ranges were broken gradually. Their Acoustic emission rose and fell rapidly with the gradual crack of the rock mass several times. The rock mass comprised of two grain size ranges was brittle crack, and its acoustic emission was strongest when the rock mass was penetrated and destroyed.
